# The long-term conditional mortality rate in older ICU patients compared to the general population

**DOI:** 10.1186/s13054-024-05147-z

**Published:** 2024-11-14

**Authors:** Anna Aronsson Dannewitz, Bodil Svennblad, Karl Michaëlsson, Miklos Lipcsey, Rolf Gedeborg

**Affiliations:** 1 Anaesthesiology and Intensive Care Medicine, Department of Surgical Sciences, Uppsala University, Uppsala, Sweden; 2 Medical Epidemiology, Department of Surgical Sciences, Uppsala University, Uppsala, Sweden

**Keywords:** Critical care, Intensive care, Comorbidity, Mortality, Older

## Abstract

**Background:**

Understanding how preexisting comorbidities may interact with a critical illness is important for the assessment of long-term survival probability of older patients admitted to the ICU.

**Material and methods:**

The mortality after a first ICU admission in patients ≥ 55 years old registered in the Swedish Intensive Care Registry was compared to age- and sex-matched individuals from the general population with a landmark after 1 year. The comparison was adjusted for age, sex, and baseline comorbidity using Cox regression.

**Results:**

The 7-year study period included 140 008 patients, of whom 23% were 80 years or older. Patients surviving the first year remained at an increased risk compared to the general population, but much of this difference was attenuated after adjustment for baseline comorbidity (HR, 1.03; 95% CI 1.02–1.04). Excluding cardio-thoracic ICU admissions, the increased risk remained slightly elevated (adjusted HR, 1.15; 95% CI 1.13–1.16). Also, the subgroup ≥ 75 years old surviving the first year returned to a mortality rate comparable to the general population (HR, 0.98; 95% CI 0.96–0.99). Stratified by admission diagnosis an increased mortality rate remained beyond the first year for acute-on-chronic respiratory failure (adjusted HR, 1.47; 95% CI 1.36–1.58) but not for other respiratory causes (adjusted HR, 1.03; 95% CI 0.99–1.07) or admission for septic shock (adjusted HR, 1.04; 95% CI 0.95–1.13). No substantial increased mortality rate was notable beyond the first year for other admission diagnoses.

**Conclusion:**

Older ICU patients that survive the first year after an ICU admission return to a mortality rate close to that of the general population having similar baseline comorbidity, but variability is seen depending on the ICU admission diagnosis.

*Trial registration* ClinicalTrials.gov ID: NCT06234709, date 02/01/2024.

**Supplementary Information:**

The online version contains supplementary material available at 10.1186/s13054-024-05147-z.

## Introduction

The development of chronic late-onset diseases is a part of aging [1], and older patients with multimorbidity are an expanding population in the intensive care unit (ICU). Age is the most influential determinant in long-term mortality following ICU discharge [2, 3], but the potential impact of comorbidity, frailty, and multimorbidity on the effects of ICU interventions on long-term outcomes remain insufficiently understood [4].

The clinical management strategy for older critically ill patients in the ICU requires a thoughtful evaluation of how the patients' acute condition interacts with the extent and severity of comorbidities and their combined impact on the long-term probability of survival. This is needed to assess the indication for ICU admission and the selection of therapeutic strategies during the ICU stay [5]. The potential impact of age and comorbidity on the patient’s prognosis is also essential in communication with the patient and family. Identifying patients who will benefit from intensive care is challenging. In a study of cancer patients admitted to the ICU, three-quarters had died within 2 years, but the survivors had good performance status [6].

Thus, at the time of ICU admission, the ability to accurately quantify the severity of different comorbidities and their potential impact on long-term survival would be valuable, especially for older patients. This could increase our understanding of how the patient’s medical history might interact with the acute condition underlying the ICU admission and the long-term mortality risk after recovery. Current severity scores assessing risk on ICU admission reflect comorbidity to some extent but may not be sufficiently discriminative [7]. A common feature of previous risk scores is to reflect each comorbidity's presence with a binary variable, disregarding potential quantitative information [8–12]. We have previously demonstrated that baseline comorbidity assessment using increased granularity of the patient’s hospital discharge history substantially improved long-term mortality prediction after ICU admission compared to the Charlson index and the Simplified Acute Physiology Score version 3 (SAPS3) score [7].

We hypothesized that the type and severity of comorbidities affect short-term and long-term mortality rates after intensive care differently and may depend on the admission diagnosis. Consequently, this study aimed to describe the long-term mortality rate after ICU admission of older patients compared to the general population. We also describe the impact of age, admission diagnosis, and seriousness of specific comorbidities on the mortality rate after ICU admission.

## Methods

### Study population

We included the first ICU admissions of patients aged 55 years and older, registered in the Swedish Intensive Care Registry (SIR) [13] from 2006 to 2012. The age restriction aimed to select a population with relevant prevalence of comorbidity. In line with the findings in a previous study [7], we ignored ICU readmissions during follow-up and considered them a part of the continuing deterioration of health following the initial ICU admission. For each ICU patient, we also included five subjects from the Swedish population. They were randomly selected from the general population alive and not hospitalized at the date of the ICU admission, and individually matched on age and sex. The regional Human Ethics Committee approved the study (Approval no 2012/197).

### Data sources

In 2012, SIR covered 92% of all ICU admissions in Sweden. SIR contains information on the characteristics of patients admitted to an ICU, reasons for admission, severity scores indicating baseline risk, comprehensive information on procedures, complications, treatment strategy, and monitoring of organ dysfunction [13].

SIR was linked to the National Patient Register [14] and the Cause of Death Register [15] using unique person identity numbers [16]. Hospital discharge diagnoses coded according to the Swedish clinical modification of the 10th revision of the International Statistical Classification of Diseases and Related Health Problems (ICD-10-SE) were retrieved from the Patient Register. Reporting to this register is mandatory and has nationwide coverage regarding in-patient care since 1987. The date of death was retrieved from the Cause of Death Register.

### Reason for ICU admission

The admission diagnoses registered in SIR based on the Acute Physiology and Chronic Health Evaluation II (APACHE II) score, or the SAPS3 score, were used to define the reason for ICU admission (eTable S1).

### Comorbidity

We used a previously described and validated method to quantify baseline comorbidity, where the time interval from the most recent hospital stay and the total length of previous hospital stays were shown to improve long-term mortality prediction compared to the Charlson comorbidity index and the SAPS3 score [7]. Hospital discharge diagnoses from in-patient care during the five years preceding the ICU admission (index) date were divided into 36 predefined comorbidity categories (eTable S2). For each comorbidity category we calculated two variables: (a) one quantifying the total length of hospital stay in days with a primary diagnosis within that category, and (b) the interval from the last hospital admission with a primary diagnosis within that category to the ICU admission date (1–6 months, 6–12 months, 1–3 years, > 3 years). The month preceding the index date was considered a grace period to reduce the inclusion of diagnoses directly related to the current hospitalization and ICU stay and not representing preexisting comorbidity. The described prevalence of comorbidity at baseline was based on both main and secondary hospital discharge diagnoses.

### Outcomes

The date of death was retrieved from the National Cause of Death Register.

### Statistical methods

Follow-up for each ICU patient and its five population-based individuals started at the day of ICU admission, the index date, and ended on the date of death or December 31, 2016, whichever came first. The length of follow-up was calculated from a ‘reverse’ Kaplan–Meier analysis [17, 18]. Survival is described using Kaplan–Meier curves with a landmark predefined to 1 year.

A propensity score based on age, sex, and all 72 comorbidity variables (two variables for each of the 36 comorbidity categories, quantifying the length of stay and the recency of previous hospital admissions within each category) was calculated from a logistic regression model. The analysis population was then restricted to the propensity score range where ICU patients and the reference individuals from the general population had overlapping distributions. The survival probability in these two groups was compared using Cox proportional hazard models estimated with inverse probability weighting for the propensity score and a robust sandwich estimator to handle the dependency from using matched subjects in the control group from the general population.

The associations between a specific comorbidity and survival probability were estimated in Cox proportional hazard models, adjusting for age, sex, and other comorbidities (eFig. S2). First, a Cox model was fitted for the association between all other comorbidity variables and survival probability. This model was then applied to the study population, and the estimated linear predictor was used to provide a summary score for the individuals' overall baseline comorbidity burden. Then, the associations between a specific comorbidity and survival probability were estimated, adjusting for age as a linear effect, sex, and other comorbidities as restricted cubic splines with 5 knots. The proportional hazards assumption was evaluated by visual inspection of -log–log plots and also tested using the R function cox.zph.

Complementary subgroup analyses were performed, where admissions to cardio-thoracic ICUs were excluded. Analyses on subpopulations defined by the admission diagnosis required an APACHE II or a SAPS3 registration. Registration of SAPS3 was only available from 2009 and onwards in SIR. In a sensitivity analysis, the subgroup analyses based on the reason for admission were restricted to ICU admissions from 2009 to 2013. To evaluate the potential consequences of this unavoidable restriction to patients with non-missing SAPS3 in this analysis, we also compared characteristics and survival rates between patients with and without SAPS3 registration.

Data management was done in SAS version 9.4, and all statistical analyses were performed using R version 3.1.3.

## Results

### Baseline characteristics

During the 7-year study period, there were 140 008 patients ≥ 55 years old with a first admission to the ICU (eFig. S1). Among them were 31 924 (23%) patients aged 80 or older (Table 1). The burden of comorbidities was notably higher among the ICU patients when compared to the age- and sex-matched controls from the general population. The most common ICU admission diagnoses were respiratory, circulatory (excluding septic shock), or neurological conditions, and the five most common comorbidity categories at baseline were hypertension, infectious diseases, ischemic heart disease, cardiac arrhythmias, and diabetes mellitus. The baseline prevalence of comorbidity and the median SAPS3 score increased with age among the ICU patients (eTable S3).

**Table 1:** Baseline characteristics of the study population, comparing ICU patients to their randomly selected age- and sex-matched individuals from the general population

	ICU patients (N = 140 008)	Individuals from the general population (N = 699 929)
Age (years)		
< 60	11% (15 713)	10% (71 326)
60–69	33% (45 764)	32% (226 006)
70–79	33% (46 607)	33% (233 600)
80–89	21% (28 936)	22% (151 597)
90 +	2% (2988)	2% (17 400)
Sex	–	–
Female	41% (56 859)	41% (283 959)
Admission diagnosis^a^		
Respiratory, other	16% (22 724)	
Circulatory (excl. septic shock)	13% (18 307)	–
Neurological	12% (16 538)	
Gastrointestinal	8% (11 573)	
Metabolic	6% (9065)	
Renal	6% (8096)	
Septic shock	3% (3847)	–
Trauma	3% (4136)	
Respiratory, acute-on-chronic	3% (4460)	
Haematological	2% (2786)	
Missing	58% (80 593)	
Comorbidities		
Hypertension	27% (37 271)	15% (103 458)
Infectious disease	21% (29 407)	10% (73 106)
Ischemic heart disease	19% (26 101)	9% (65 355)
Cardiac arrhythmias	16% (21 929)	9% (60 511)
Diabetes mellitus	14% (19 039)	6% (44 151)
Bone/muscle disease	13% (17 636)	8% (58 783)
Congestive heart failure	13% (17 561)	5% (34 884)
Injury	12% (17 179)	8% (56 283)
Neurological disease	12% (16 520)	8% (54 886)
Chronic pulmonary disease	11% (15 043)	4% (27 951)
Tumour non-metastatic	10% (13 643)	6% (38 643)
Cerebrovascular disease	9% (12 165)	6% (39 330)
Peripheral vascular disease	8% (10 591)	3% (17 704)
Other anaemias	7% (9521)	3% (17 795)
Valvular disease	7% (9128)	1% (8617)
Renal disease	6% (8088)	2% (15 272)
Other endocrine disease	4% (6246)	2% (15 552)
Alcohol abuse	4% (5926)	1% (9401)
Rheumatic/autoimmune disease	4% (5094)	2% (11 379)
Fluid balance disorder	3% (4754)	1% (9440)
Depression	3% (4610)	2% (10 883)
Hepatic disease	2% (3237)	1% (3681)
Pulmonary circulation disorder	2% (2678)	1% (5656)
Tumour metastatic	2% (2537)	1% (6123)
Drug abuse	2% (2492)	0% (3480)
Haematological malignancy	2% (2279)	1% (3935)
Poisoning	2% (2208)	1% (3636)
Haematological disease	2% (2199)	1% (3593)
Obesity	1% (1794)	0% (3288)
Blood loss anaemia	1% (1550)	1% (3587)
Psychoses	1% (1412)	1% (3659)
Malnutrition	1% (783)	0% (1801)
Transplantation-related disorder	1% (720)	0% (711)
Coagulopathy	0% (671)	0% (1384)
Immunodeficiency	0% (203)	0% (317)

^a^Admission diagnoses registered in the Swedish Intensive Care register are based on the APACHE II score for the early part of the study period, and the SAPS3 score that is available from the year 2009 onwards. See supplementary eTable S1 for harmonisation of admission diagnoses. Note that one patient can have multiple admission diagnoses registered

### Overall survival compared with the general population

**Fig. 1:**
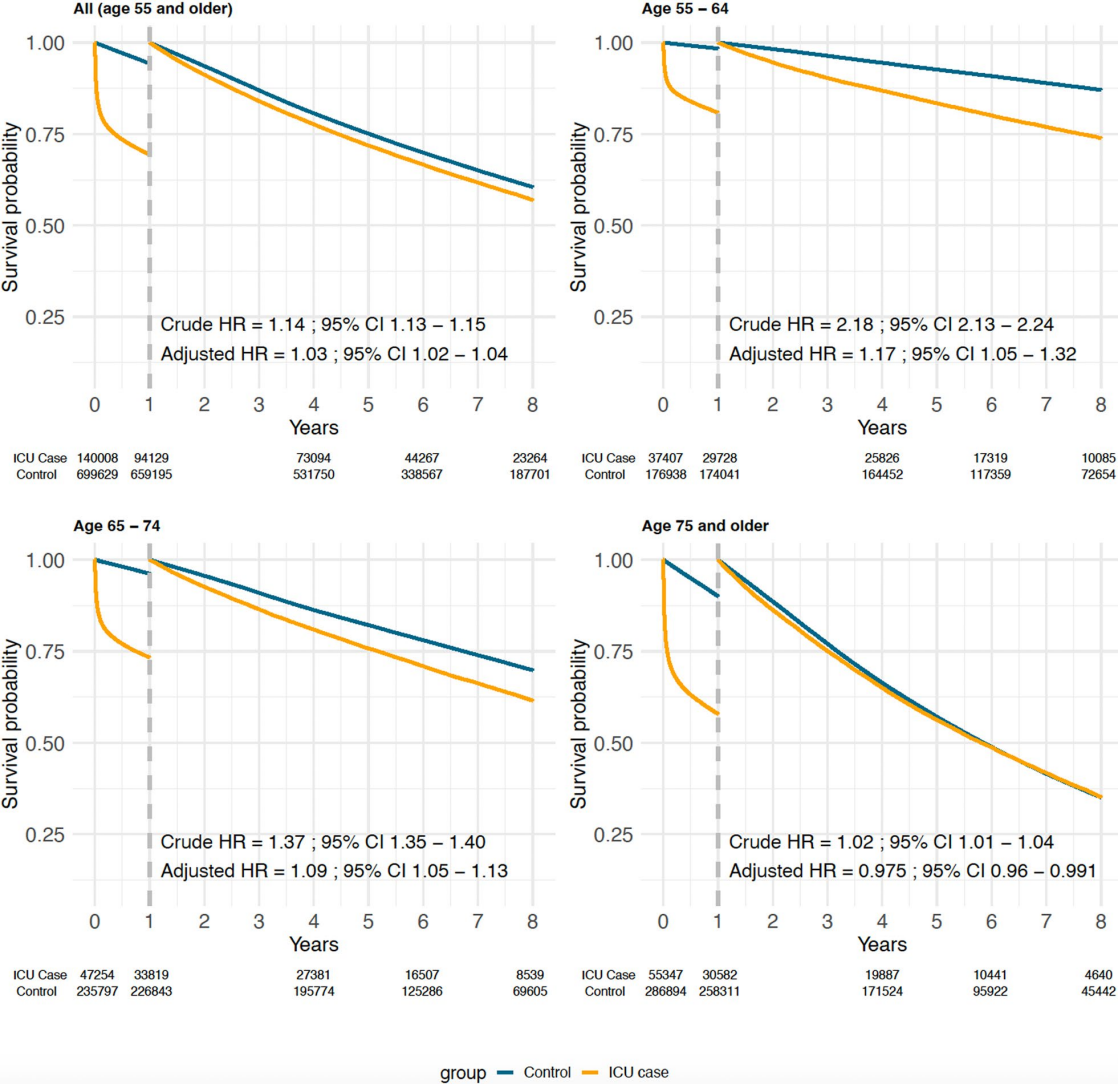
Landmark analysis of survival probability after admission to intensive care. Survival described by Kaplan–Meier curves is compared to age- and sex-matched individuals from the general population, and separately for different age groups. After the first year of follow-up, the survival analysis has been reset to follow those patients that survived the first year after ICU admission. Hazard ratios with 95% confidence intervals (CI) have been estimated in Cox proportional hazards models adjusted for age, sex, and comorbidity

Being admitted to the ICU was overall associated with a 28-day mortality risk of 20.7% and a 1-year mortality risk of 32.8%, compared to a 1-year mortality risk of 5.8% in the age- and sex-matched individuals from the general population (adjusted HR, 5.8; 95% CI 5.6–5.9) (eTable S4). ICU patients surviving the first year continued to have a higher mortality rate compared with the general population (crude HR, 1.14; 95% CI 1.13–1.15), but this difference was to a large degree attenuated after adjustment for baseline comorbidity (adjusted HR, 1.03; 95% CI 1.02–1.04) (Fig. 1).

When stratified for age, a slightly elevated mortality risk remained after 1 year in 55–64-year-olds compared with the general population, also after adjusting for comorbidity (adjusted HR, 1.17; 95% CI, 1.05–1.32) (Fig. 1). This increased risk diminished with increasing age and was not seen in patients aged 75 years and older (adjusted HR, 0.98; 95% CI, 0.96–0.99).

When admissions to cardio-thoracic intensive care units were excluded from the analysis, the overall risk remained elevated after 1 year, also after adjusting for comorbidity (adjusted HR, 1.15; 95% CI, 1.13–1.16) (eFig. S3). This remaining increase in mortality rate was also seen in this restricted analysis population, which was low in patients aged 75 years and older (adjusted HR, 1.08; 95% CI, 1.06–1.10).

**Fig. 2:**
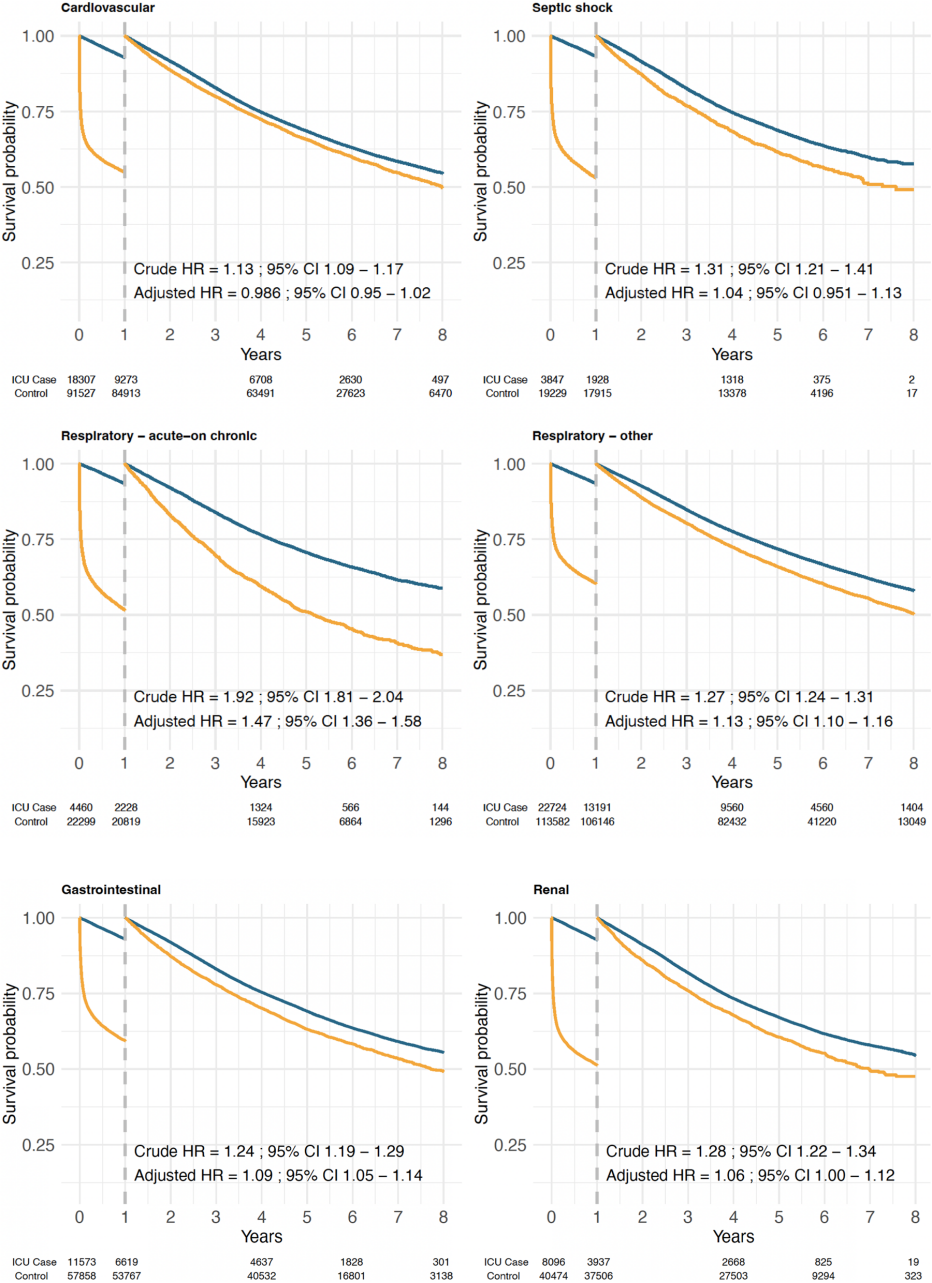

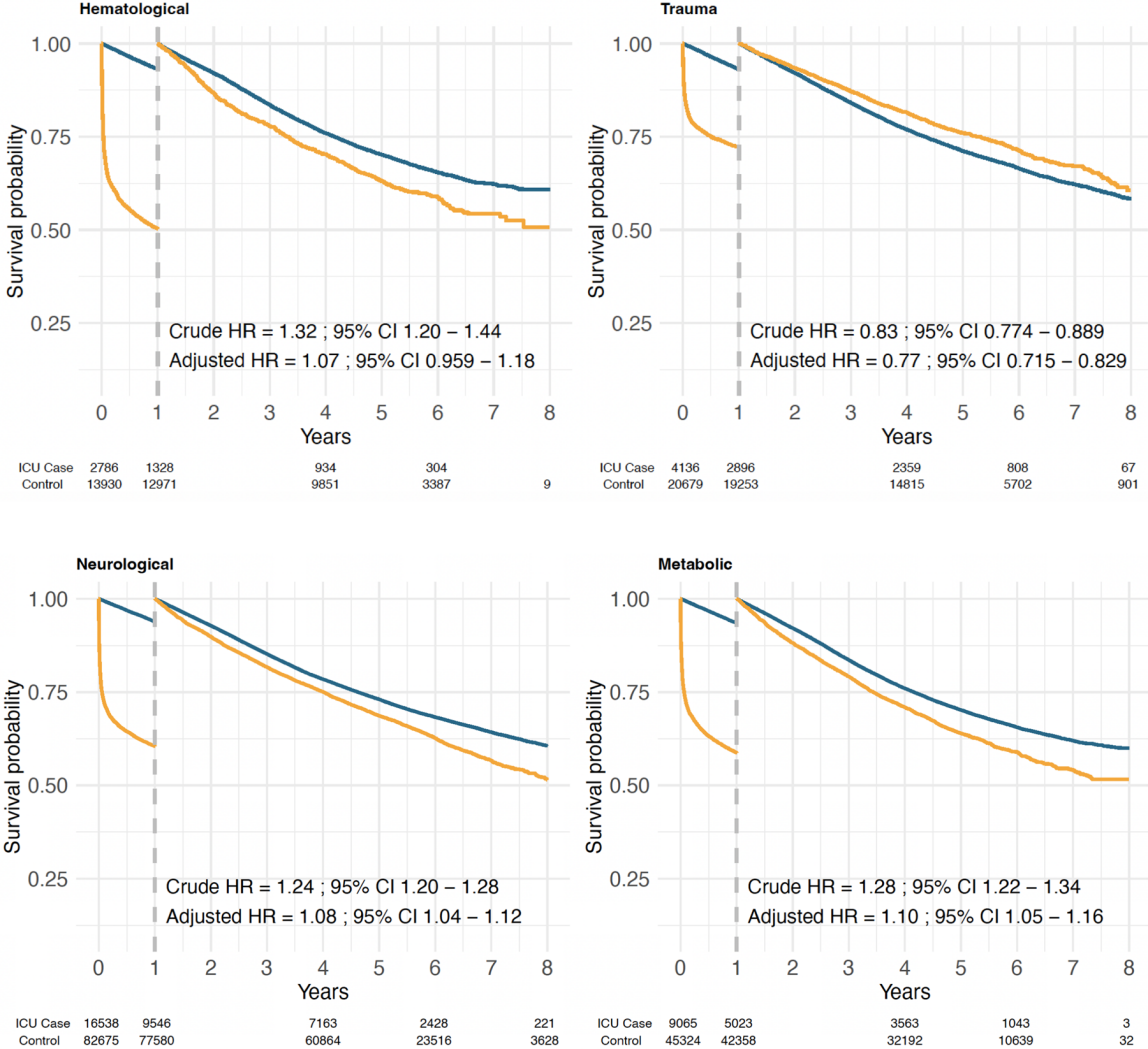
Landmark analysis of survival probability after admission to intensive care. Survival described by Kaplan–Meier curves is compared to age- and sex-matched individuals from the general population, and separately for different reasons for ICU admission. After the first year of follow-up, the survival analysis has been reset to follow those patients that survived the first year after ICU admission. Hazard ratios with 95% confidence intervals (CI) have been estimated in Cox proportional hazards models adjusted for sex, age, and comorbidity

When the study population was stratified by the reason for ICU admission, no increase in mortality rate remained beyond the first year after ICU admissions for cardiovascular reasons (adjusted HR, 0.98; 95% CI 0.95–1.02) or septic shock (adjusted HR, 1.04; 95% CI 0.951–1.13) (Fig. 2). Patients admitted for acute-on-chronic respiratory failure, however, had the most notable remaining increased risk after 1 year (adjusted HR, 1.47; 95% CI 1.36–1.58) (Fig. 2). A weaker remaining association was seen after ICU admissions for other respiratory causes (adjusted HR, 1.13; 95% CI 1.10–1.16), but this association was largely attenuated when the analysis was restricted to the later period with patients having SAPS3 scores registered (adjusted HR, 1.03; 95% CI 0.99–1.07) (eFig. S4). Similar patterns were seen for other admission diagnoses except for trauma, where the mortality rate was increased during the first year (adjusted HR, 4.6; 95% CI 4.3–5.0), but the patients that survived the first year had a 23% lower mortality rate than individuals from the general population (adjusted HR, 0.77; 95% CI 0.715–0.829) (Fig. 2 and eTable S5).

### Specific baseline comorbidities and survival

#### Hypertension

Hypertension was the most common comorbidity identified in 27% of previous hospital admissions in ICU patients (Table 1). However, only a recent prior hospital admission with hypertension as the main diagnosis was weakly associated with a mortality rate after adjustment for other comorbidities (eFig. S5).

#### Infection

**Fig. 3:**
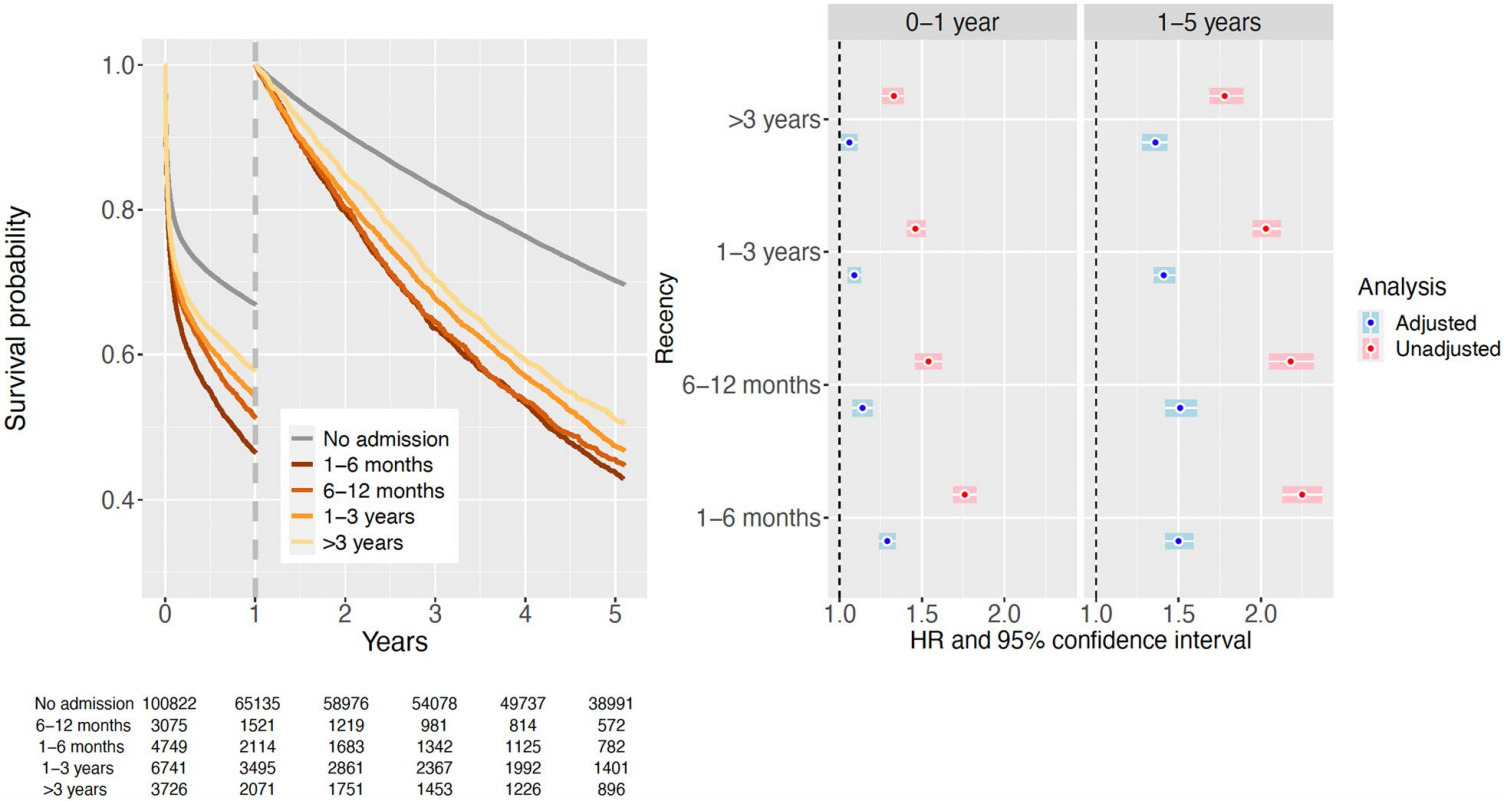
Landmark survival analysis described by Kaplan–Meier curves (left panel), stratified for recency of a previous hospital admission with infection as the main discharge diagnosis. After the first year of follow-up, the survival analysis has been reset to follow those patients that survived the first year after ICU admission. Hazard ratios with 95% confidence intervals (right two panels) have been estimated from Cox proportional hazards models separately for each time period, comparing an unadjusted analysis with an analysis adjusted for sex, age, and other comorbidities

A prior hospital admission for infection was associated with an increased mortality rate, with a gradual increase in mortality rate the more recent the hospital admission (Fig. 3 and eTable S6). This association was stronger in patients surviving the first year after ICU admission, being most notable for patients having a prior hospital admission for infection within 6 months (crude HR, 2.25; 95% CI 2.13–2.37). This association was attenuated after adjustment for other comorbidities (adjusted HR, 1.50; 95% CI 1.42–1.59).

#### Cardiac disease

**Fig. 4:**
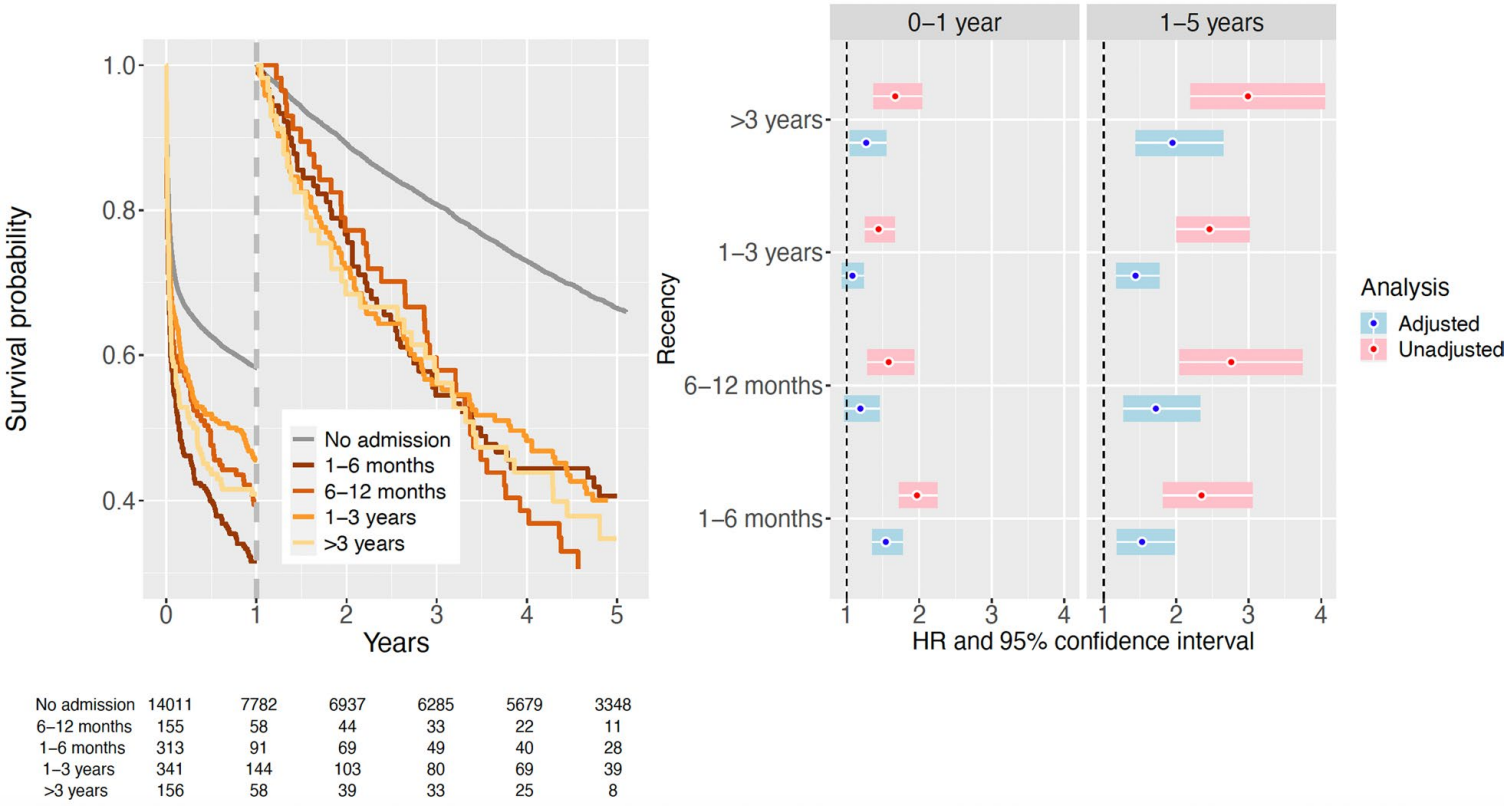
Subgroup analysis restricted to ICU admissions with a SAPS3 reason for admission classified as respiratory but excluding patients with acute-on-chronic respiratory failure. Landmark survival analysis described by Kaplan–Meier curves (left panel), stratified for recency of a previous hospital admission with heart failure as the main discharge diagnosis. After the first year of follow-up, the survival analysis has been reset to follow those patients that survived the first year after ICU admission. Hazard ratios (HR) with 95% confidence intervals (CI) (right two panels) have been estimated from Cox proportional hazards models separately for each time period, comparing an unadjusted analysis with an analysis adjusted for sex, age, and other comorbidities

After adjustment for other comorbidities, prior hospital admission for ischemic heart disease was not an independent risk factor for mortality (eFig. S6a, eTable S6), especially when admissions to cardio-thoracic ICUs were excluded (eFig. S6b, eTable S6). The recency of prior hospital admission for arrhythmia appeared to have a reverse trend, with more recent admissions having a weaker association with mortality rate (eFig. S7, eTable S6). Adjustment for other comorbidities largely removed these associations. In the 8578 ICU patients that had a prior hospital admission for heart failure, this was a risk factor for mortality rate both during the first year of follow-up and among those surviving 1 year after ICU admission (eFig. S8, eTable S6). There was, however, no apparent relation with the recency of the prior heart failure admission, and the association was notably attenuated by adjustment for other comorbidity. In subgroups restricted to admission for respiratory (other than acute-on-chronic) (Fig. 4) or circulatory reasons (eFig. S9) there were similar patterns (eTable S6).

There were 16 495 ICU patients with a previous hospital admission for chronic pulmonary disease. The recency of this admission was strongly associated with the mortality rate during the first year and among those surviving the first year (eFig. S10, eTable S6). In subgroups restricted to admission for circulatory (eFig. S11) or respiratory (other than acute-on-chronic) reasons (eFig. S12), there was a similar pattern but with weaker associations during the first year after ICU admission (eTable S6).

### Missing SAPS3 score

Among the 78 053 ICU admissions from 2009 and onwards, when SAPS3 had been introduced in SIR, 34 368 patients (44%) did not have a SAPS3 registration. The proportion with missing SAPS3 decreased over time (eTable S7). Compared to those with SAPS3 registered, a substantially higher proportion (34% vs. 0%) were admissions to cardio-thoracic ICUs, slightly fewer were women, and they tended to have shorter ICU length of stay (eTable S7). The prevalence of comorbidity appeared essentially comparable. When comparing mortality rates, patients with missing SAPS3 seemed to have a substantially higher survival probability (eFig. S13).

### Propensity score overlap and proportional hazards assumption

The propensity score distributions in the main analysis with the comparison to the general population showed good overlap, with only 9 subjects of 839 637 removed from the analysis due to non-overlap (eFig. S14).

An evaluation of the proportional hazards assumptions for the main comparison shows acceptable compliance for the follow-up after the 1-year landmark (eFig. S15). The HRs presented for the first year after the ICU admission, with comparisons against the general population, should as evident from the Kaplan–Meier curves be interpreted with caution given the very high immediate mortality after ICU admission. These HRs still provide an average relative mortality rate, but it should be recognised that the ratio of hazards clearly varies over time before the 1-year landmark.

## Discussion

In this large nationwide population-based study, older patients admitted to the ICU and who survived the first year after admission had a slight increase in mortality rate in the following years compared with the general population. This difference was, however, strongly attenuated by adjustment for baseline comorbidity, leaving only a weak association that was least notable in the highest age groups. The reason for ICU admission had a variable impact on the long-term mortality rate, with the most apparent increase seen in patients admitted for acute-on-chronic respiratory insufficiency.

In ICU patients 75 years and older, survival within the first year after intensive care admission was slightly above 50%. Still, after the 1-year landmark, survival was mainly comparable to the general population's age- and sex-matched controls. The difference compared to the general population was almost negligible in the oldest age group. Behind this age-related pattern, there are likely complex interactions between the type and severity of medical conditions leading to ICU admission, prevalent comorbidity, and other patient-related factors such as general frailty, but also external factors such as bed-space availability and regional clinical practice patterns influencing the decision to admit the patient to the ICU.

Attempts to compare the long-term mortality risk in survivors of critical illness to that of the normal population have been made previously. In a retrospective observational cohort study of 2104 adult patients admitted to the ICU of a teaching hospital in Glasgow from 1985 to 1992 the risk of mortality in survivors of critical illness matches that of the normal population after four years [19]. No adjustment for baseline comorbidity was, however, attempted.

In a recent prospective cohort study of emergency ICU admissions in 3920 patients ≥ 80 years of age, 64% of patients had died after 6 months [20]. This is largely comparable to the mortality rate among patients 75 years and older in our study. Patient frailty was found predictive of mortality with a 6-month follow-up in this population of very old patients. No comparison group was available to contextualise the mortality risk, and comorbidity was measured as a count of comorbid conditions. This has been shown to be a suboptimal measure of comorbidity [7, 21, 22]. The use of a count of medications taken daily before admission has also been outperformed by other drug comorbidity measures [23, 24]. The impact of comorbid conditions may therefore have been underestimated also in this study.

Patient frailty is a complex multisystemic syndrome that has been associated with adverse outcomes after intensive care [25]. However, the direct impact on the effectiveness of ICU interventions on outcomes of intensive care remains uncertain [4]. Patients with frailty admitted to the ICU have a more significant number of comorbidities and are generally older [26]. An interaction between comorbidity and frailty has high face validity but remains poorly studied. In the prospective FORECAST study [25] baseline comorbidity was captured with the Carlson index, a suboptimal measure of comorbidity [7, 21, 22].

Our study population included patients admitted to general ICUs and cardiothoracic ICUs. This must be considered when interpreting the results. When the SAPS3 prognostic score was developed, many cardiac surgery patients were included in the development dataset [27]. SAPS3 has nevertheless been shown to perform less well in this patient category, and other risk adjustment instruments have been used instead [28]. The reason for this could be that the cardio-thoracic ICUs predominantly admit patients for postoperative care and that patient selection for cardiac surgery strongly impacts long-term survival probability [29]. Therefore, analyses restricted to admissions to general ICUs may be of particular relevance for the overall interpretation of our results.

Knowledge of how the individual patient’s age and history of comorbidity interact with the acute condition underlying the ICU admission and the long-term survival probability compared to the general population after recovery is essential background information for selecting appropriate treatment strategies. This understanding is also relevant for the communication with patient and family, and it would be of value if some reassurance could be provided that if the patient survives the first year after the ICU stay, life expectancy 1 year after ICU admission will return to what would have been expected based on age and preexisting comorbidities. It should, however, be acknowledged that the results in this study are conditional on the selection of patients for ICU admission. We have no knowledge related to patients that were potentially eligible for intensive care but for some reason were not admitted to an ICU. It may also be relevant to consider the independent impact of specific comorbidities.

The strength and pattern of the association between the recency of a prior admission for a specific comorbid condition and mortality rate were variable, depending on the type of comorbidity. The more recent a previous hospitalization for chronic pulmonary disease was, the stronger the association with mortality rate was, both before and after the 1-year landmark. Heart failure, another chronically progressive comorbidity, was also clearly associated with mortality rate after adjustment for other comorbidity, but without any apparent relation to recency for the prior hospital admission for heart failure.

The recency of a prior admission for ischemic heart disease is considered an established risk factor for mortality after non-cardiac surgery [30]. A similar pattern would be expected after ICU admission. No such association could, however, be observed in our study. After adjustment for other comorbidities, a prior hospitalization for ischemic heart disease was not associated with mortality rate, neither before nor after the 1-year landmark. New treatment strategies for acute coronary syndrome have substantially improved the outcome of ischemic heart disease over the years, and this may partly explain this finding [31]. It should be recognized that the association between recency of prior hospital admission for a comorbidity and mortality rate following an ICU stay may also be variable depending on differences in healthcare utilization between countries and regions over time [32].

The observation that the strength of association with survival probability is variable depending on the specific comorbidity condition studied is expected. The mortality rate associated with an exacerbation of chronic obstructive pulmonary disease (COPD) is high, especially if requiring ICU admission [22]. Some associations observed are not as expected. A history of hypertension or a previous hospitalization for infection may not be expected to impact the survival probability associated with an ICU admission. Still, a recent prior hospital admission for any of these conditions appeared to confer an increased mortality rate also after adjustment for other comorbidities.

Some strengths of our study are the large population-based study population with almost complete coverage of all Swedish ICUs, comprehensive data collection, long-term follow-up, and individuals selected from the general population. The quantification of comorbidity is based on a model shown to outperform conventional comorbidity measures [7]. These measures may still be suboptimal and cause residual confounding. An example could be chronic obstructive pulmonary disease, where physiological measures of lung function or other functional measures could provide further risk stratification. Differences in coding practices and healthcare system characteristics between countries are also expected to limit generalizability and hamper international comparisons [33, 34]. Other factors related to patient frailty and survival probability, such as functional status, cognitive function, socioeconomic status, and lifestyle, were not included in our study and could result in residual confounding. They may be essential determinants for long-term outcomes, but they were outside the scope of the present study [35–37]. Further studies to explore potential interaction between comorbidity and very high age would also be relevant [38].

The fact that follow-up is only available until December 2016 can be considered a limitation. It should, however, be acknowledged that inclusion of data from 2019 inevitably would have compromised the interpretation of results because of the COVID-19 pandemic. When we using SIR compare ICU casemix and mortality in 2008 with 2023, they are very similar [39]. The mean age on ICU admission was 54.5 years in 2008 and 57.5 years in 2023. The median max, Sequential Organ Failure Assessment (SOFA) score, was 9 both in 2011 (first year available) and in 2023. The median length of stay was 0.97 days in 2008 and 1.09 days in 2023. Mortality after 180 days was 22.0% in 2008 and 21.9% in 2023. During the pandemic years with COVID-19, however, the pattern was different due to the large number of people treated in intensive care for COVID-19 and the limited resources available. To include these years in the current study would notably complicate the interpretation and limit generalizability of the results. It should also be recognized that the risk adjustment instrument SAPS3 was developed on data collected from consecutively admitted patients between 14 October and 15 December 2002 [27].

One key limitation of the study is that admission to the ICU is preceded by an individual patient assessment, and this may involve selection mechanisms that are time-dependent and context sensitive [40]. Since we do not have any information on which patients that were potentially eligible but not actually admitted to the ICU, the consequences of such selection mechanisms remain elusive. The results should be considered with this limitation in mind.

The analyses stratified for admission diagnosis are hampered by the change from APACHE II to SAPS3 during the study period and the large proportion of patients without a registered score. Because the scoring is predominantly missing from cardio-thoracic ICU admissions and patients with higher survival probability and considering the difficulty in harmonizing APACHE II and SAPS3 admission diagnoses, the analyses restricted to when SAPS3 is available are considered to be the most relevant.

Another potential limitation is the selection of a 1-year landmark in the survival analyses. It is unclear how long after an ICU stay the course of the acute illness can affect mortality. For randomized ICU intervention trials, long-term mortality is also considered an important outcome measure. The European Medicines Agency guideline on clinical investigation of medical products for treating sepsis recommends that longer-term mortality data should support the primary outcome measure with a minimum of 6 months follow-up [41]. Similar recommendations are given for treatments targeting patients with acute respiratory distress syndrome [42]. To isolate long-term effects from the acute phase, we therefore pre-specified the landmark to 1 year. This selection may have influenced the results but has a clinical rationale.

## Conclusion

Older patients admitted to the ICU have a higher baseline burden of comorbidity, higher severity of illness, and consequently a higher mortality rate during the first year. Those who survive the first year after an ICU admission return to close to the mortality rate of the general population having similar comorbidity. Also, ICU patients 75 years and older returned to a mortality rate largely comparable to the general population. There was variability related to the reason for ICU admission, with the most apparent remaining long-term increase in mortality rate seen in patients admitted for acute-on-chronic respiratory insufficiency.

## Supplementary Information

Supplementary Material 1.

## Data Availability

The data that support the findings of this study are available from the Swedish Intensive Care Register, National Patient Register and the Cause of Death Register but restrictions apply to the availability of these data, which were used under license for the current study, and so are not publicly available. Data are however available from the authors upon reasonable request and with permission of the registers.
