# Supplementary material for: The long-term conditional mortality rate in older ICU patients compared to the general population
Source: Crit Care. 2024 Nov 14;28:368. doi: 10.1186/s13054-024-05147-z (PMC11566578; doi:10.1186/s13054-024-05147-z)

# The long-term conditional mortality rate in older ICU patients compared to the general population --

# Supplementary online-only material

**eFigure S1.** Flow chart describing selection of the study population.


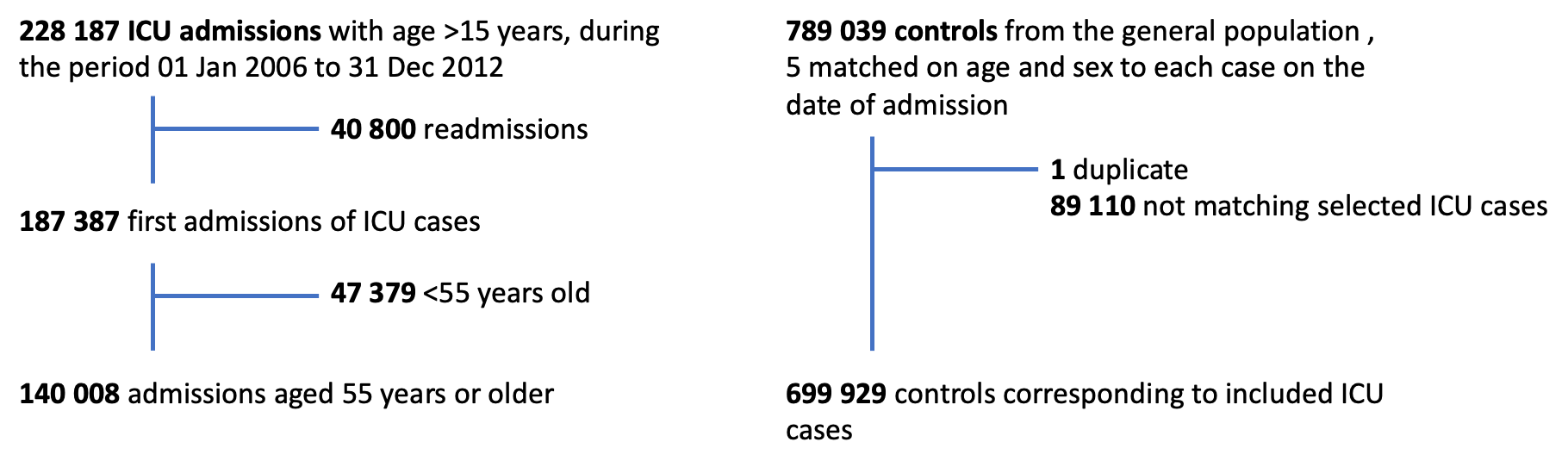


**eTable S1**. Categorisation of admission diagnoses based on APACHE and SAPS3 as registered in the Swedish Intensive Care Register (SIR). Registration of SAPS3 was introduced in 2009 and therefore only available from the later part of the study period.

| Area | APACHE - Reason for admission | SAPS3 - Reason for admission | Categorisation of reason for ICU admission in the study |
| --- | --- | --- | --- |
| Cardiovascular | Cardiac arrest | Cardiac arrest | Circulatory (excl. septic shock) |
|  | Acute myocardial infarction |  |  |
|  |  | Anaphylactic shock |  |
|  | Other cardiovascular disease | Other cardiovascular |  |
|  |  | Mixed/undefined shock |  |
|  |  | Hypovolemic/haemorrhagic shock |  |
|  |  | Hypovolemic/non-haemorrhagic shock |  |
|  | Cardiogenic shock | Cardiogenic shock |  |
|  | Cardiac failure |  |  |
|  |  | Anaphylactic shock |  |
|  |  | Chest pain |  |
|  | Hypertension | Hypertensive crisis |  |
|  | Arrhythmia | Arrhythmia |  |
|  |  | Cardiovascular failure without shock |  |
| Cardiovascular |  | Septic shock | Septic shock |
| Hepatic |  | Hepatic failure | Hepatic |
|  | Cholecystitis/cholangitis |  |  |
|  |  | Other hepatic reason |  |
| Gastrointestinal |  | Acute abdomen | Gastrointestinal |
|  |  | Gastrointestinal bleeding |  |
|  |  | Pancreatitis |  |
|  | Gastrointestinal obstruction |  |  |
|  | Gastrointestinal perforation/rupture |  |  |
|  | Gastrointestinal inflammatory disease |  |  |
|  | Other gastrointestinal disease | Other gastrointestinal reason |  |
| Cardiovascular |  | Septic shock | Septic shock |
| Respiratory | Chronic obstructive pulmonary disease | Acute pulmonary failure on chronic pulmonary failure | Respiratory, acute-on-chronic |
| Respiratory |  | Acute pulmonary failure, ARDS | Respiratory, other |
|  | Aspiration pneumonia |  |  |
|  | Bacterial/viral pneumonia |  |  |
|  | Pulmonary infection |  |  |
|  | Parasitic pneumonia |  |  |
|  | Pulmonary oedema (non-cardiogenic) |  |  |
|  | Respiratory arrest |  |  |
|  | Respiratory neoplasm |  |  |
|  | Asthma |  |  |
|  | Pulmonary embolism |  |  |
|  | Mechanical airway obstruction |  |  |
|  | Other respiratory disease | Other respiratory reason |  |
| Trauma |  | Trauma | Trauma |
|  | Multiple trauma (excl. head injury) |  |  |
|  | Burn |  |  |
| Neurological |  | Focal neurological deficit | Neurological |
|  |  | Intracranial expansion |  |
|  | Head trauma (with/without multiple trauma) |  |  |
|  | Subdural/epidural hematoma |  |  |
|  | Intracerebral bleeding |  |  |
|  |  | Epileptic seizures |  |
|  |  | Reduced consciousness |  |
|  | Subarachnoid hemorrhage |  |  |
|  | Other neurological disease |  |  |
| Renal |  | Renal failure | Renal |
|  |  | Postrenal kidney failure |  |
|  |  | Prerenal kidney failure |  |
|  | Other renal disease | Other renal reason |  |
| Haematological |  | Bleeding, DIC | Haematological |
|  |  | Severe haemolysis |  |
|  |  | Other haematological reason |  |
| Metabolic |  | Hypo/hyperthermia | Metabolic |
|  |  | Hypo/hyperglycemia |  |
|  |  | Acid-base or electrolyte disorder |  |
|  |  | Other metabolic reason |  |

**eTable S2.** Operational definitions of comorbidity categories using ICD-9 or ICD-10. The definitions according to Elixhauser et al have been expanded and new categories added [*Elixhauser A, Steiner C, Harris DR, Coffey RM, (1998) Comorbidity measures for use with administrative data. Med Care 36: 8-27*].

|  | Comorbidity category | ICD-9 | ICD-10 |
| --- | --- | --- | --- |
| 1 | Congestive heart failure (CHF) | 398, 393A, 402A, 402X, 404A, 404B, 404X, 425, 428, 429 | I13, I42, I50, I51 |
| 2 | Ischemic heart disease (IHD) | 410-414 | I20-I25 |
| 3 | Valvular disease (Valv) | 394-397, 421, 424 | I05-I08, I091, I33-I39 |
| 4 | Cardiac arrythmias (Ary) | 427, 427 | I44-I49, R00 |
| 5 | Pulmonary circulation disorders (Pulmcirc) | 415-417 | I26-I28 |
| 6 | Hypertension (HT) | 401-405 | I12, I15, I109, I119 |
| 7 | Cerebrovascular disease (CVD) | 430-438 | I60-I69 |
| 8 | Peripheral vascular disease (Perivasc) | 440-447, 452, 453, 456 | I70-I82, I85-I89, R029 |
| 9 | Neurologic disease (Neurol) | 290, 293, 294, 310, 320-359, 780A, 781C, 781D, 784D, 784F | R40, R41, R47, R48, G00-G99, F00-F09, R25-R29, R296, R298 |
| 10 | Chronic pulmonary disease (CPD) | 490-505, 506E, 506X, 508B, 515-517, 519B, 519E, 519W | D860, D862, E662, E840, J40-J67, J684, J701, J703, J84, J850, J953, J961, J969, J98, J99 |
| 11 | Infectious disease (Inf) | 001-066, 070B, 071-078, 079A-079D, 079W, 079X, 080-139, 460-466, 473, 480-491, 510, 511, 590, 595, 597, 790H, 790W, 998F, 999D, | A00-B09, B25-B99, B159, J00-J32, J851-J869, M00-M01, M726, N300, N390, T880 |
| 12 | Diabetes (Diab) | 250 | E10-E14 |
| 13 | Other endocrine disorders (Xendo) | 251-259 | E00-E07, E15-E35, E89 |
| 14 | Renal disease (Renal) | 403, 404, 580-589 | N00-N12, N14-N19, V42A, V45B, V56, R34, R392, Z992 |
| 15 | Hepatic disease (Hepatic) | 070A, 070C-070X, 456A, 456B, 570-573 | B150, B16-B18, I85, K70-K77, V42H |
| 16 | Immune deficiencies incl. HIV (Immundef) | 173, 279, 079J | D89, B20-B24, D80-D84, V02J |
| 17 | Hematological malignancies (Hemomal) | 200-208, | C81-C96, D477-D479 |
| 18 | Other hematological disease (Hemodis) | 288, 289 | D45, D46, D471-D473, D69-D77 |
| 19 | Solid tumour without metastases (tumournomet) | 140-195 | C00-C76, Z510, Z511, Z926 |
| 20 | Metastatic cancer (Tumourmet) | 196-199 | C77-C80, C979 |
| 21 | Rheumatic disease (Collagen) | 279N, 710, 713D, 713H, 714, 720, 725, 729A-729E, | M05-M09, M30-M35 |
| 22 | Coagulopathy (Coag) | 286, 287 | D65-D68 |
| 23 | Obesity (Obese) | 278 | E65, E66 |
| 24 | Nutritional deficiences (Nutr) | 260-269 | E40-E64, E90 |
| 25 | Fluid and electrolyte disorders (Fluid) | 276 | E86, E87, R631 |
| 26 | Blood loss and anemia (Bleed) | 285B | D629 |
| 27 | Deficiency and other anemia (Anemia) | 280-284, 285A, 285W, 285X | D50-D61, D64 |
| 28 | Alcohol abuse (Alco) | 291, 303, 305A, 790D | F10, K70, K852, R780, Y90, Y91, Z502, Z721 |
| 29 | Drug abuse (Drug) | 292, 304, 305B, 305X | F11-F19, R781-R786, Z503, Z722, |
| 30 | Psychoses (Psycho) | 295, 297-299, 780B | F20-F29, R44 |
| 31 | Affective disorders (Affect) | 296, 311 | F30-F39 |
| 32 | Other psychiatric disorders (Xpsych) | 300-302, 307-309, 312-319 | F40-F99, Z504 |
| 33 | Transplantation (Tx) | 996W | V42, T86, Z940-Z944 |
| 34 | Bone or muscle disease (Bonemusc) | 715-738 | M12-M25, M40-M83, M858, M859, M86-M90, M99 |
| 35 | Injury (Injury) | 800-959, 990-994, 995F | S00-T35, T66-T79, T90-T98 |
| 36 | Poisoning (Intox) | 960-989 | T36-T65 |

**eFigure S2**. Directional acyclic graphs (DAG) describing the assumed associations between age (Panel A) and severity of specific comorbidity (Panel B) with the outcome all-cause death.

**Panel A**:

**
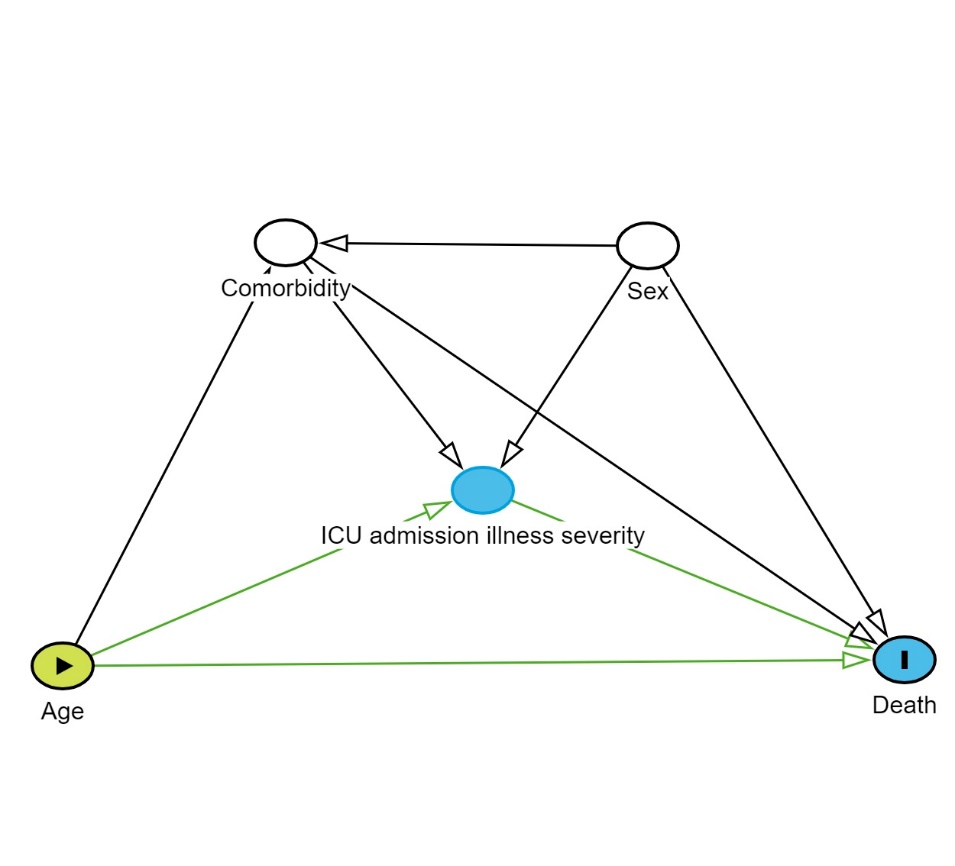
**

**Panel B**:


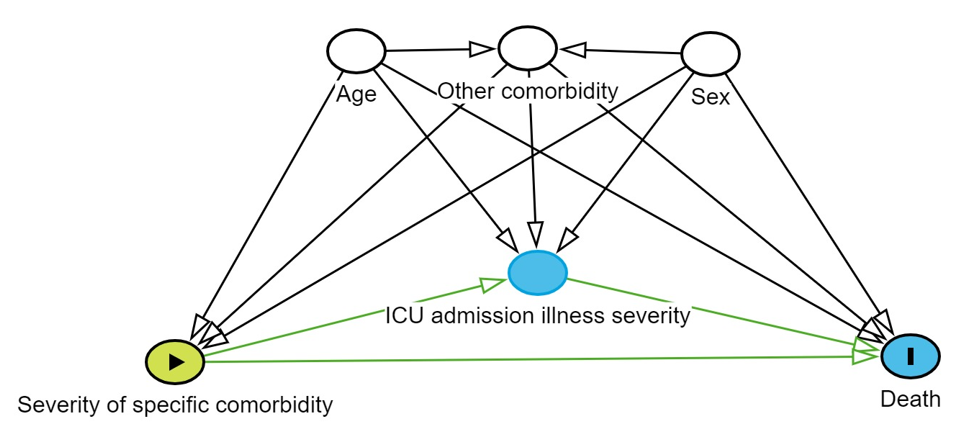


**eTable S3**. Baseline characteristics of 140 008 ICU patients, comparing baseline characteristics stratified by age.

|  | Age categories | | | | |
| --- | --- | --- | --- | --- | --- |
|  | <60 years  (N=15 713) | 60-69 years  (N=45 764) | 70-79 years  (N=46 607) | 80-89 years  (N=28 936) | ≥90 years  (N=2988) |
| **Age** (years), median (interquartile range) | 57 (56-58) | 65 (62-67) | 75 (72-77) | 83 (81-86) | 91 (90-93) |
| **Sex**, % (n) |  |  |  |  |  |
| Female | 36% (5649) | 36% (16 442) | 40% (18 835) | 49% (14 139) | 60% (1794) |
| **ICU type**, % (n) |  |  |  |  |  |
| Thoracic | 16% (2458) | 17% (7954) | 18% (8226) | 8% (2235) | 1% (22) |
| General | 84% (13 255) | 83% (37 810) | 82% (38 381) | 92% (26 701) | 99% (2966) |
| Burn | 0% (0) | 0% (0) | 0% (0) | 0% (0) | 0% (0) |
| **Reason for ICU admission**^a^, % (n) |  |  |  |  |  |
| Circulatory (excl. septic shock) | 9% (1468) | 12% (5290) | 14% (6433) | 16% (4638) | 16% (478) |
| Septic shock | 2% (298) | 3% (1223) | 3% (1345) | 3% (906) | 3% (75) |
| Gastrointestinal | 7% (1029) | 8% (3474) | 8% (3761) | 10% (2969) | 11% (340) |
| Neurological | 13% (2014) | 13% (5776) | 11% (5251) | 11% (3171) | 11% (326) |
| Renal | 4% (555) | 5% (2308) | 6% (2962) | 7% (2067) | 7% (204) |
| Respiratory, acute-on-chronic | 2% (332) | 3% (1353) | 4% (1734) | 3% (966) | 3% (75) |
| Respiratory, other | 15% (2424) | 16% (7251) | 16% (7308) | 18% (5182) | 19% (559) |
| Haematological | 2% (259) | 2% (915) | 2% (958) | 2% (598) | 2% (56) |
| Metabolic | 6% (916) | 6% (2955) | 6% (2979) | 7% (2017) | 7% (198) |
| Trauma | 4% (570) | 3% (1436) | 2% (1085) | 3% (869) | 6% (176) |
| Missing | 61% (9567) | 59% (26 855) | 58% (27 132) | 54% (15 491) | 52% (1548) |
| **SAPS3 score**, median (interquartile range) | 47 (39-58) | 53 (44-64) | 59 (50-70) | 64 (55-73) | 62 (55-71) |
| **Type of comorbidity present at baseline** |  |  |  |  |  |
| Alcohol abuse | 11% (1652) | 7% (3005) | 2% (1086) | 1% (182) | 0% (1) |
| Blood loss anemia | 1% (104) | 1% (393) | 1% (541) | 2% (452) | 2% (60) |
| Bone/muscle disease | 8% (1257) | 10% (4638) | 14% (6540) | 16% (4740) | 15% (461) |
| Cardiac arrhythmias | 6% (955) | 10% (4562) | 18% (8244) | 25% (7315) | 29% (853) |
| Cerebrovascular disease | 4% (652) | 6% (2932) | 10% (4554) | 13% (3632) | 13% (395) |
| Chronic pulmonary disease | 6% (982) | 9% (4318) | 13% (5890) | 12% (3590) | 9% (263) |
| Coagulopathy | 0% (49) | 0% (202) | 0% (231) | 1% (170) | 1% (19) |
| Congestive heart failure | 5% (820) | 8% (3742) | 14% (6451) | 20% (5814) | 25% (734) |
| Depression | 5% (735) | 3% (1570) | 3% (1376) | 3% (848) | 3% (81) |
| Diabetes | 10% (1553) | 13% (6084) | 15% (7150) | 14% (3937) | 11% (315) |
| Drug abuse | 4% (578) | 2% (1110) | 1% (616) | 1% (182) | 0% (6) |
| Fluid balance disorder | 3% (410) | 3% (1385) | 3% (1574) | 4% (1233) | 5% (152) |
| Hematological disease | 2% (236) | 2% (810) | 2% (730) | 1% (387) | 1% (36) |
| Hematological malignancy | 1% (216) | 2% (841) | 2% (807) | 1% (390) | 1% (25) |
| Hepatic disease | 6% (869) | 3% (1516) | 1% (635) | 1% (207) | 0% (10) |
| Hypertension | 15% (2434) | 22% (10 241) | 30% (14 070) | 33% (9567) | 32% (959) |
| Immunodeficiency | 0% (37) | 0% (98) | 0% (49) | 0% (18) | 0% (1) |
| Infectious disease | 14% (2250) | 17% (7758) | 21% (9976) | 29% (8367) | 35% (1056) |
| Injury | 10% (1532) | 10% (4446) | 12% (5360) | 18% (5068) | 26% (773) |
| Ischemic heart disease | 10% (1573) | 15% (6723) | 21% (9985) | 25% (7097) | 24% (723) |
| Malnutrition | 0% (75) | 0% (228) | 1% (247) | 1% (204) | 1% (29) |
| Neurological disease | 9% (1475) | 11% (4812) | 12% (5562) | 15% (4220) | 15% (451) |
| Obesity | 2% (310) | 2% (828) | 1% (511) | 1% (145) | 0% (0) |
| Other anemias | 4% (673) | 5% (2413) | 7% (3226) | 10% (2849) | 12% (360) |
| Other endocrine disease | 3% (453) | 3% (1573) | 5% (2219) | 6% (1791) | 7% (210) |
| Peripheral vascular disease | 5% (799) | 7% (3127) | 9% (4007) | 8% (2445) | 7% (213) |
| Poisoning | 3% (439) | 2% (732) | 1% (586) | 1% (409) | 1% (42) |
| Psychoses | 2% (309) | 1% (603) | 1% (347) | 1% (146) | 0% (7) |
| Pulmonary circulation disorders | 1% (170) | 2% (716) | 2% (1033) | 2% (693) | 2% (66) |
| Renal disease | 4% (652) | 5% (2246) | 6% (2971) | 7% (2026) | 6% (193) |
| Rheumatic/autoimmune disease | 2% (311) | 3% (1293) | 4% (1900) | 5% (1483) | 4% (107) |
| Transplantation-related disorder | 1% (164) | 1% (397) | 0% (145) | 0% (14) | 0% (0) |
| Tumour metastatic | 2% (333) | 2% (1070) | 2% (812) | 1% (301) | 1% (21) |
| Tumour non-metastatic | 8% (1183) | 10% (4412) | 11% (4961) | 10% (2855) | 8% (232) |
| Valvular disease | 4% (635) | 5% (2363) | 8% (3689) | 8% (2306) | 5% (135) |

^a^ Admission diagnoses registered in the Swedish Intensive Care register are based on the APACHE score for the early part of the study period, and the SAPS3 score that is available from the year 2009 onwards. Se supplementary eTable S1 for harmonisation of admission diagnoses. Note that one patient can have multiple admission diagnoses registered.

**eFigure S3.** Subgroup analysis excluding ICU admissions from thoracic intensive care. Landmark analysis of survival probability after admission to intensive care. Survival described by Kaplan-Meier curves is compared to age- and sex-matched control groups from the general population, and separately for different age groups. Hazard ratios with 95% confidence intervals (CI) have been estimated in Cox proportional hazards models adjusted for age, sex and comorbidity.


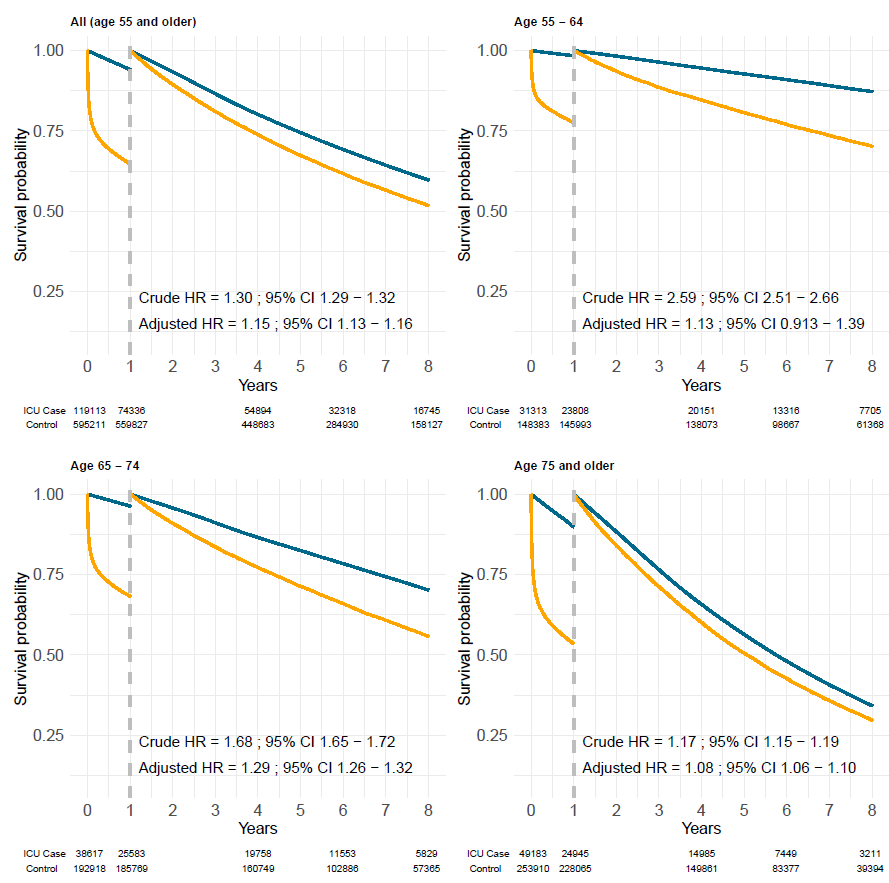


**eFigure S4**. Landmark analysis of survival probability after admission to intensive care, stratified by the reason for ICU admission and restricted to 2009-2012 when SAPS3 scoring is available in the Swedish intensive care register. Survival described by Kaplan-Meier curves is compared to age- and sex-matched control groups from the general population. Hazard ratios with 95% confidence intervals (CI) have been estimated in Cox proportional hazards models adjusted for sex, age, and comorbidity.


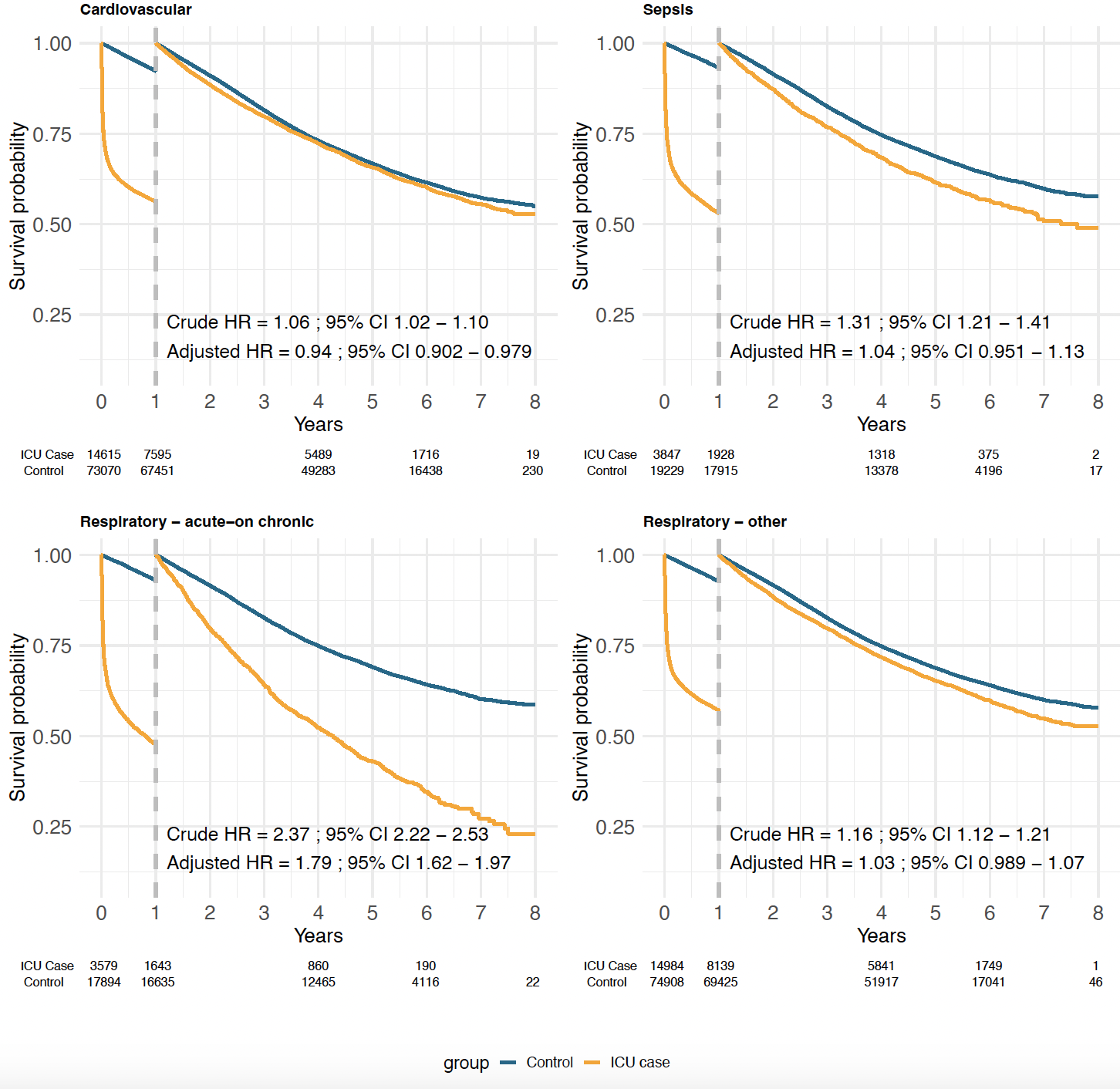


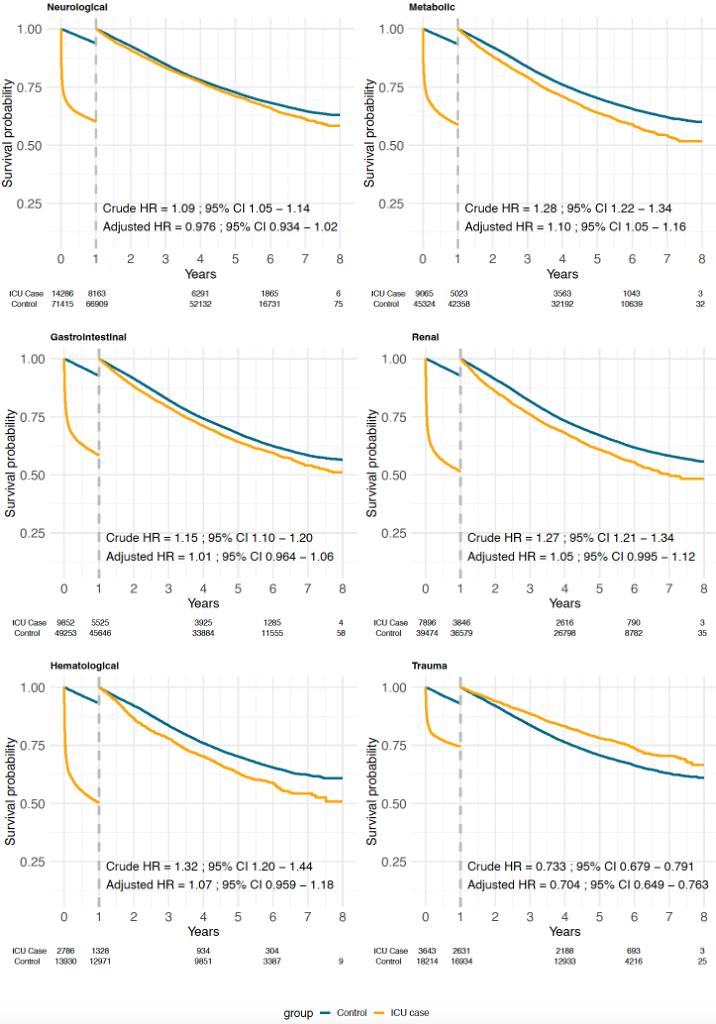


**eTable S4.** Comparison of survival probability during the first year after ICU admission. Patients admitted to the ICU are compared to controls from the general population, matched on age and sex. Cox proportional hazards regression with results presented as hazard ratios (HR) with 95% confidence intervals (CI). Unadjusted analyses as well as analyses adjusted for age, sex, and comorbidity are presented.

|  |  | Unadjusted analyses | | | Adjusted analyses | | |
| --- | --- | --- | --- | --- | --- | --- | --- |
| Admission diagnosis ICU | Group | Total number of ICU patients | Total number of deaths | HR (95% CI) | Total number of ICU patients | Total number of deaths | HR (95% CI) |
| Any | ICU Case | 140 008 | 45 879 | 7.2 (7.1 to 7.3) | 140 001 | 45 874 | 5.8 (5.6 to 5.9) |
|  | Controls | 699 929 | 40 436 | Reference | 699 927 | 40 435 | Reference |
| Circulatory (excl. septic shock) | ICU Case | 14 615 | 7 020 | 9.2 (8.9 to 9.6) | 14 606 | 7 013 | 8 (7.7 to 8.3) |
|  | Controls | 73 070 | 5 619 | Reference | 73 063 | 5 618 | Reference |
| Septic shock | ICU Case | 3 847 | 1 919 | 10.9 (10.1 to 11.7) | 3 815 | 1 899 | 8 (7.2 to 8.7) |
|  | Controls | 19 229 | 1 314 | Reference | 19 197 | 1 310 | Reference |
| Gastrointestinal | ICU Case | 9 852 | 4 327 | 8.4 (8 to 8.8) | 9 824 | 4 316 | 6.8 (6.5 to 7.2) |
|  | Controls | 49 253 | 3 607 | Reference | 49 234 | 3 605 | Reference |
| Nervous system | ICU Case | 14 286 | 6 123 | 9.5 (9.2 to 9.9) | 14 281 | 6 121 | 8.5 (8.1 to 8.8) |
|  | Controls | 71 415 | 4 506 | Reference | 71 373 | 4 497 | Reference |
| Renal | ICU Case | 7 896 | 4 050 | 10.7 (10.2 to 11.2) | 7 874 | 4 040 | 8.2 (7.8 to 8.8) |
|  | Controls | 39 474 | 2 895 | Reference | 39 455 | 2 891 | Reference |
| Respiratory, acute-on-chronic | ICU Case | 3 579 | 1 936 | 11.9 (11.1 to 12.8) | 3 559 | 1 924 | 8.2 (7.1 to 9.3) |
|  | Controls | 17 894 | 1 259 | Reference | 17 817 | 1 247 | Reference |
| Respiratory, other | ICU Case | 14 984 | 6 845 | 8.9 (8.6 to 9.3) | 14 975 | 6 837 | 7.5 (7.2 to 7.8) |
|  | Controls | 74 908 | 5 483 | Reference | 74 896 | 5 480 | Reference |
| Hematology | ICU Case | 2 786 | 1 458 | 11.6 (10.7 to 12.6) | 2 746 | 1 433 | 8.1 (6.8 to 9.8) |
|  | Controls | 13 930 | 959 | Reference | 13 892 | 955 | Reference |
| Metabolic | ICU Case | 9 065 | 4 042 | 9.6 (9.2 to 10.1) | 9 056 | 4 039 | 7.6 (7.1 to 8.1) |
|  | Controls | 45 324 | 2 966 | Reference | 45 294 | 2 961 | Reference |
| Trauma | ICU Case | 3 643 | 1 012 | 4.8 (4.5 to 5.2) | 3 636 | 1 008 | 4.6 (4.3 to 5) |
|  | Controls | 18 214 | 1 280 | Reference | 18 138 | 1 260 | Reference |

**eTable S5.** Survival probability for ICU patients that survived the first year after ICU admission. Patients admitted to the ICU are compared to controls from the general population, matched on age and sex. Cox proportional hazards regression with results presented as hazard ratios (HR) with 95% confidence intervals (CI). Unadjusted analyses as well as analyses adjusted for age, sex, and comorbidity are presented.

|  |  | Unadjusted analyses | | | Adjusted analyses | | |
| --- | --- | --- | --- | --- | --- | --- | --- |
| Admission diagnosis ICU | Group | Total number of ICU patients | Total number of deaths | HR (95% CI) | Total number of ICU patients | Total number of deaths | HR (95% CI) |
| Any | ICU Case | 94 129 | 38 248 | 1.14 (1.13 to 1.15) | 94 127 | 38 246 | 1.03 (1.02 to 1.04) |
|  | Controls | 659 493 | 250 377 | Reference | 659 492 | 250 376 | Reference |
| Circulatory (excl. septic shock) | ICU Case | 7 595 | 2 854 | 1.06 (1.02 to 1.1) | 7 593 | 2 852 | 0.94 (0.9 to 0.98) |
|  | Controls | 67 451 | 24 595 | Reference | 67 445 | 24 592 | Reference |
| Septic shock | ICU Case | 1 928 | 790 | 1.31 (1.21 to 1.41) | 1 916 | 781 | 1.04 (0.95 to 1.13) |
|  | Controls | 17 915 | 6 106 | Reference | 17 887 | 6 092 | Reference |
| Gastrointestinal | ICU Case | 5 525 | 2 146 | 1.15 (1.1 to 1.2) | 5 508 | 2 137 | 1.01 (0.96 to 1.06) |
|  | Controls | 45 646 | 16 168 | Reference | 45 629 | 16 157 | Reference |
| Nervous system | ICU Case | 8 163 | 2 594 | 1.09 (1.05 to 1.14) | 8 160 | 2 594 | 0.98 (0.93 to 1.02) |
|  | Controls | 66 909 | 19 941 | Reference | 66 876 | 19 920 | Reference |
| Renal | ICU Case | 3 846 | 1 626 | 1.27 (1.21 to 1.34) | 3 834 | 1 621 | 1.05 (1 to 1.12) |
|  | Controls | 36 579 | 13 146 | Reference | 36 564 | 13 139 | Reference |
| Respiratory, acute-on-chronic | ICU Case | 1 643 | 1 010 | 2.37 (2.22 to 2.53) | 1 635 | 1 004 | 1.79 (1.62 to 1.97) |
|  | Controls | 16 635 | 5 612 | Reference | 16 570 | 5 566 | Reference |
| Respiratory, other | ICU Case | 8 139 | 3 080 | 1.16 (1.12 to 1.21) | 8 138 | 3 079 | 1.03 (0.99 to 1.07) |
|  | Controls | 69 425 | 23 653 | Reference | 69 416 | 23 646 | Reference |
| Hematology | ICU Case | 1 328 | 529 | 1.32 (1.2 to 1.44) | 1 313 | 520 | 1.07 (0.96 to 1.18) |
|  | Controls | 12 971 | 4 228 | Reference | 12 937 | 4 208 | Reference |
| Metabolic | ICU Case | 5 023 | 1 957 | 1.28 (1.22 to 1.34) | 5 017 | 1 955 | 1.10 (1.05 to 1.16) |
|  | Controls | 42 358 | 13 742 | Reference | 42 333 | 13 730 | Reference |
| Trauma | ICU Case | 2 631 | 641 | 0.73 (0.68 to 0.79) | 2 628 | 640 | 0.70 (0.65 to 0.76) |
|  | Controls | 16 934 | 5 373 | Reference | 16 878 | 5 336 | Reference |

**eFigure S5**. Landmark survival analysis described by Kaplan-Meier curves (left panel), stratified for recency of a previous hospital admission with hypertension as the main discharge diagnosis. Hazard ratios with 95% confidence intervals (right two panels) have been estimated from Cox proportional hazards models separately for each time period, comparing an unadjusted analysis with an analysis adjusted for sex, age, and other comorbidities.


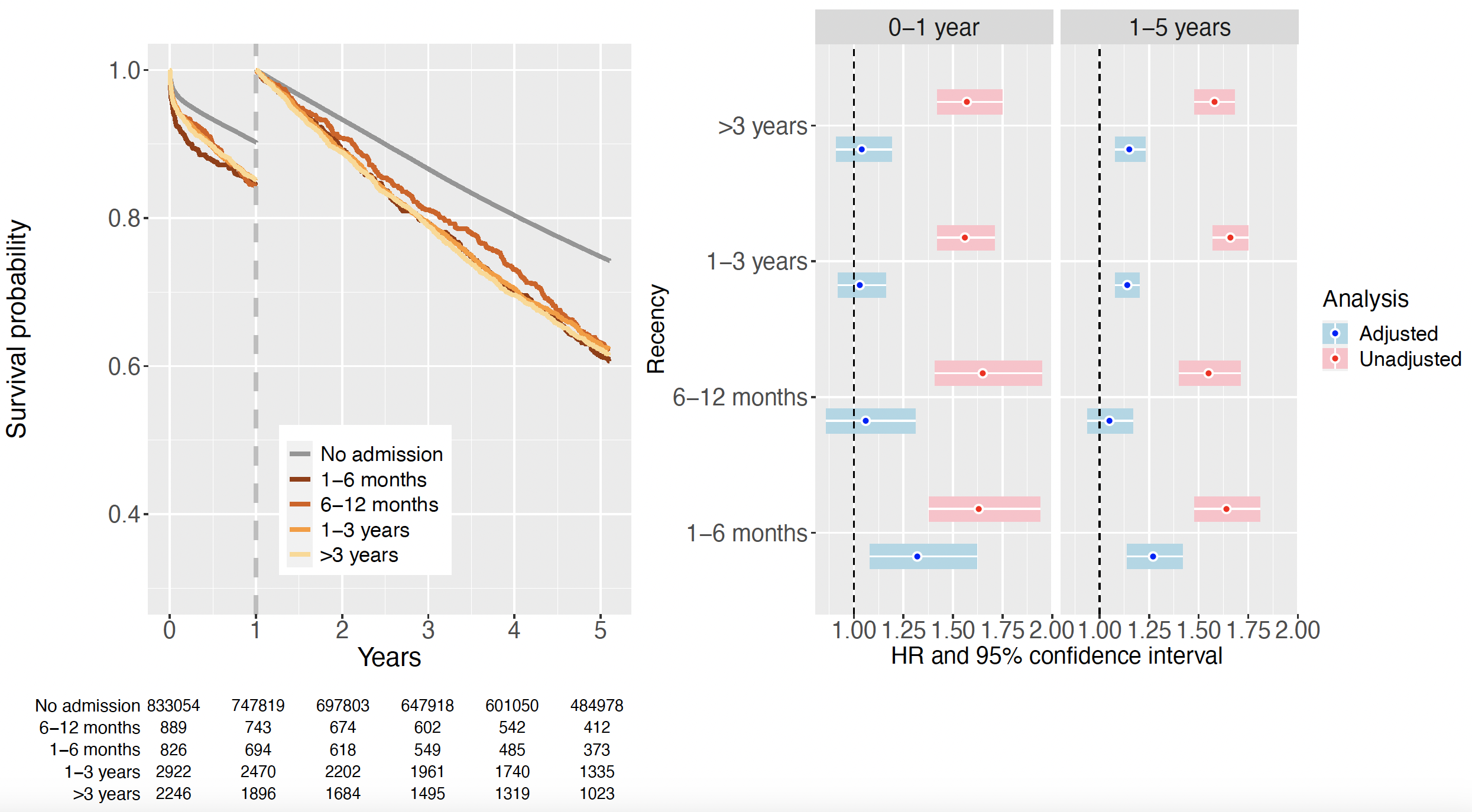


**eFigure S6a**. Landmark survival analysis described by Kaplan-Meier curves (left panel), stratified for recency of a previous hospital admission with ischemic heart disease as the main discharge diagnosis. Hazard ratios with 95% confidence intervals (right two panels) have been estimated from Cox proportional hazards models separately for each time period, comparing an unadjusted analysis with an analysis adjusted for sex, age, and other comorbidities.
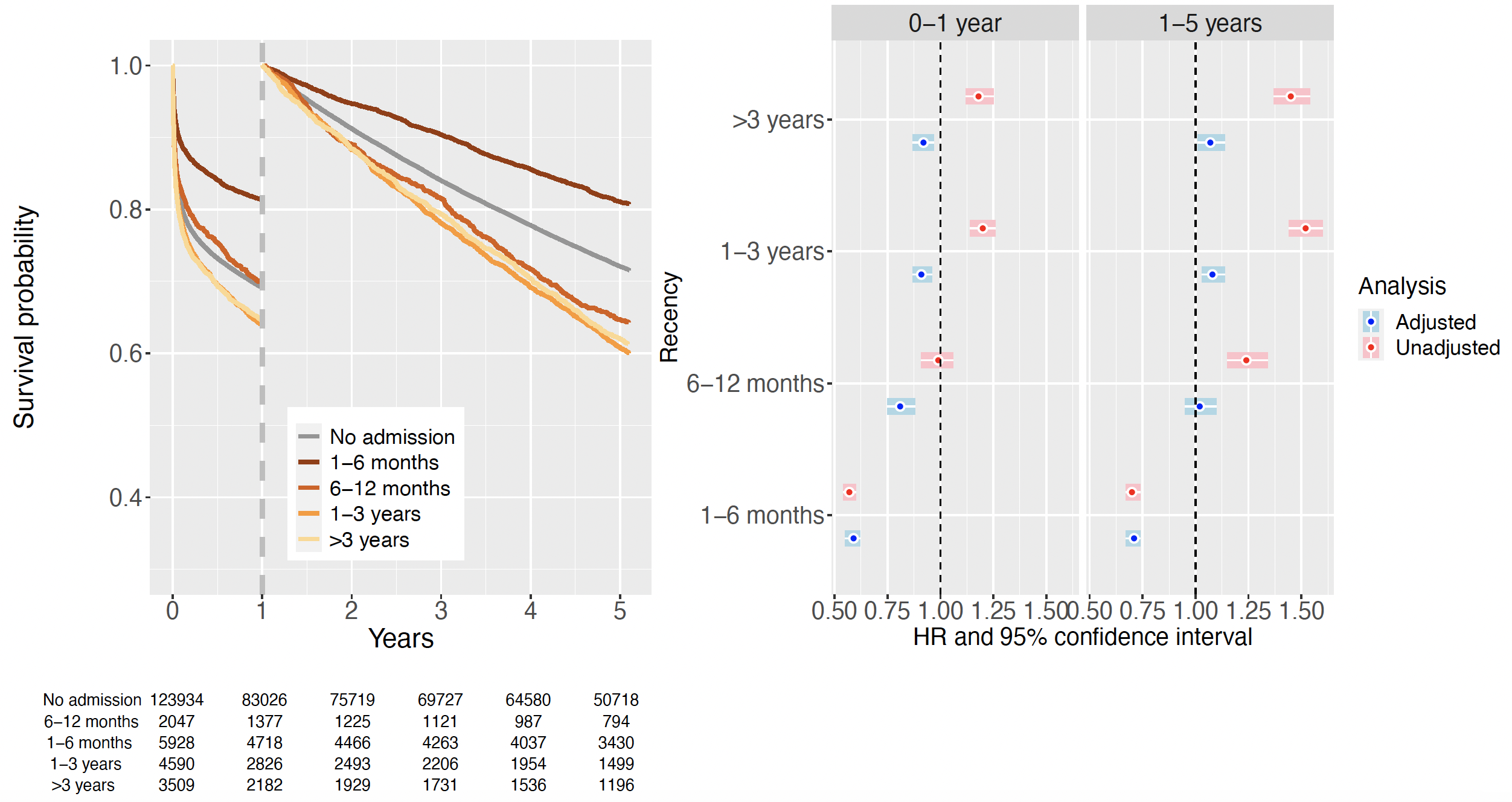


**eFigure S6b**. Subgroup analysis restricted to admissions to general ICUs (excluding thoracic ICUs). Landmark survival analysis described by Kaplan-Meier curves (left panel), stratified for recency of a previous hospital admission with ischemic heart disease as the main discharge diagnosis. Hazard ratios (HR) with 95% confidence intervals (CI) (right two panels) have been estimated from Cox proportional hazards models separately for each time period, comparing an unadjusted analysis with an analysis adjusted for sex, age, and other comorbidities.


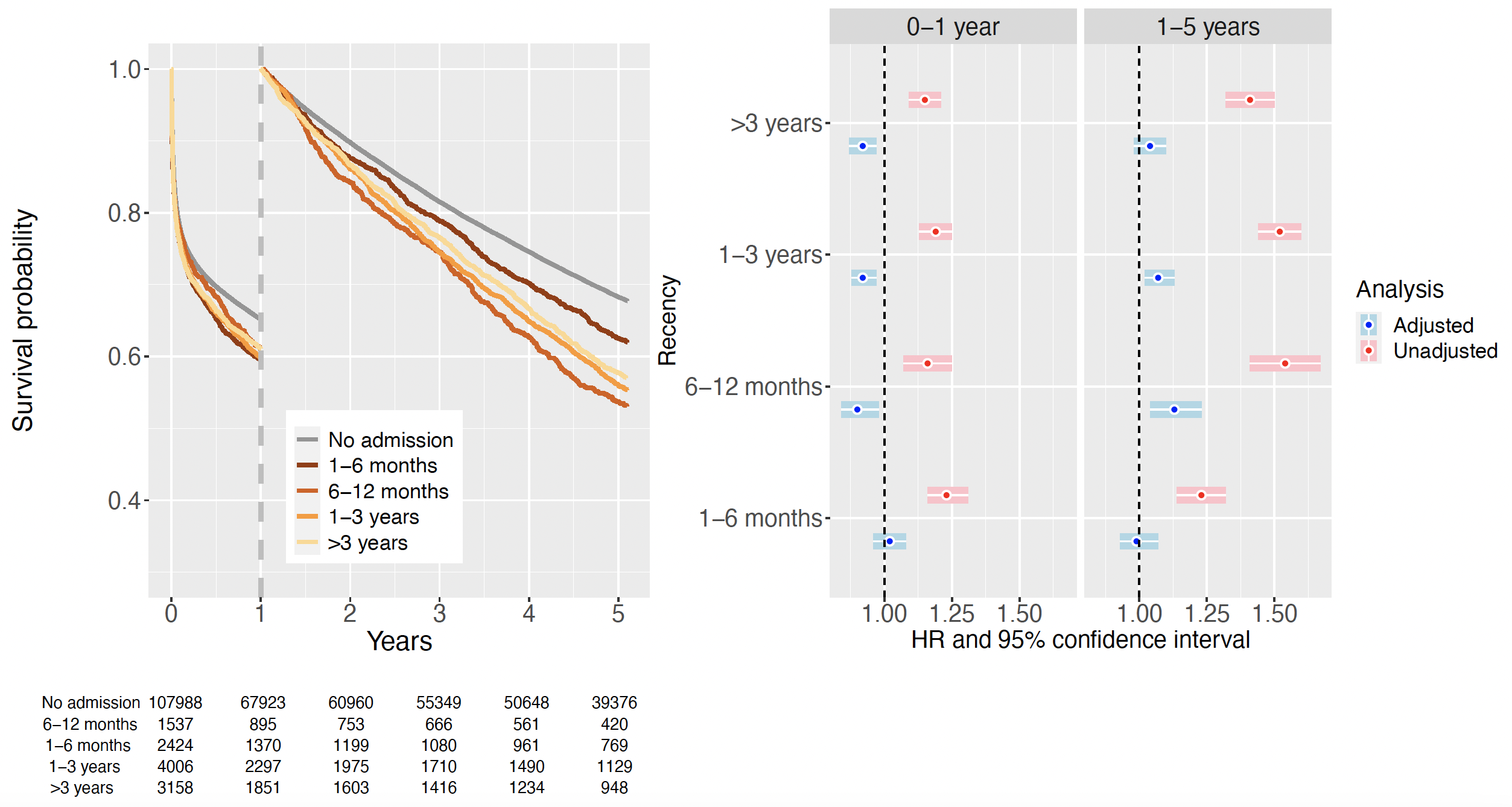


**eFigure S7**. Landmark survival analysis described by Kaplan-Meier curves (left panel), stratified for recency of a previous hospital admission with arrythmia as the main discharge diagnosis. Hazard ratios with 95% confidence intervals (right two panels) have been estimated from Cox proportional hazards models separately for each time period, comparing an unadjusted analysis with an analysis adjusted for sex, age, and other comorbidities.


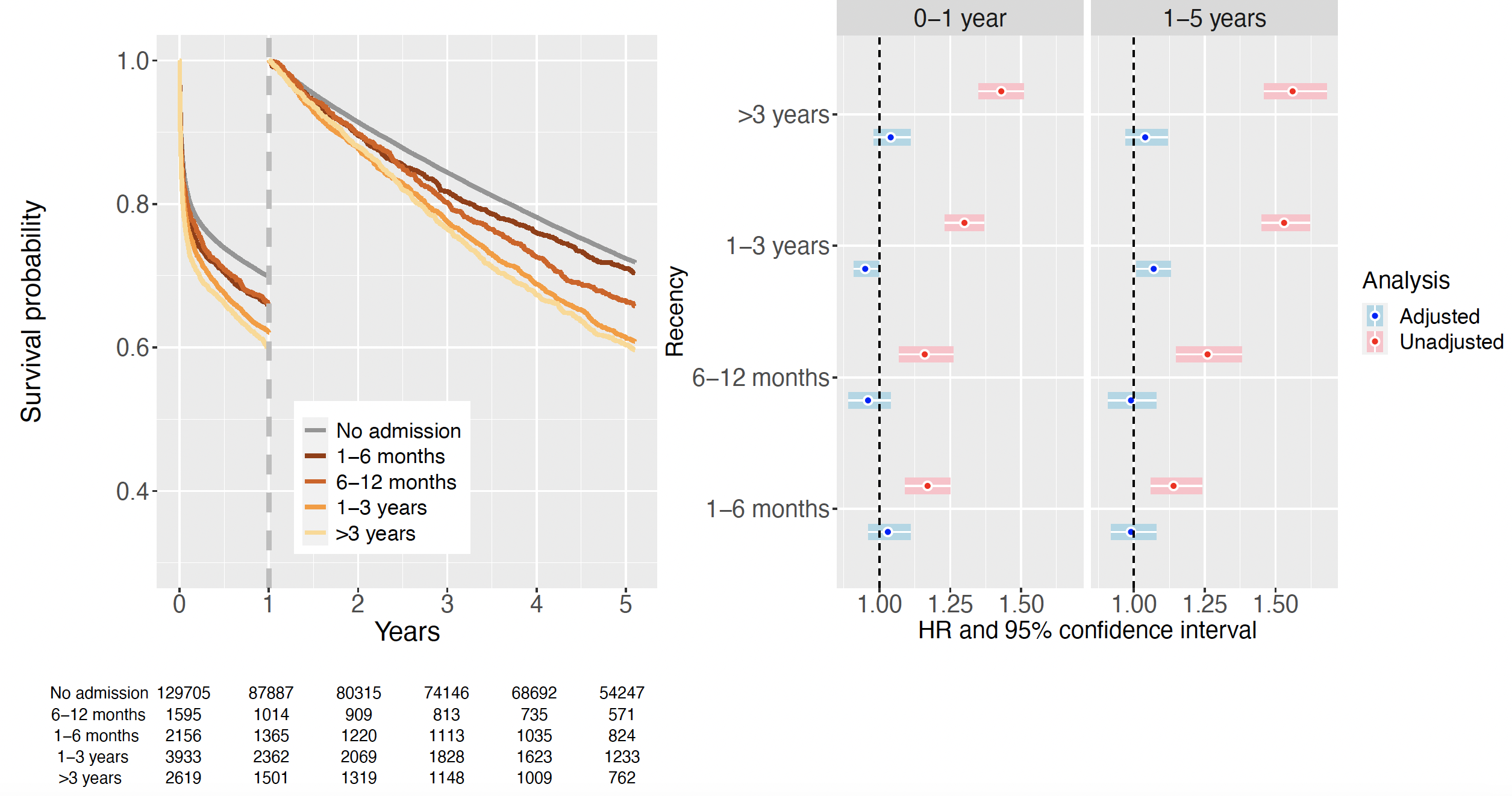


**eFigure S8**. Landmark survival analysis described by Kaplan-Meier curves (left panel), stratified for recency of a previous hospital admission with heart failure as the main discharge diagnosis. Hazard ratios with 95% confidence intervals (right two panels) have been estimated from Cox proportional hazards models separately for each time period, comparing an unadjusted analysis with an analysis adjusted for sex, age, and other comorbidities.


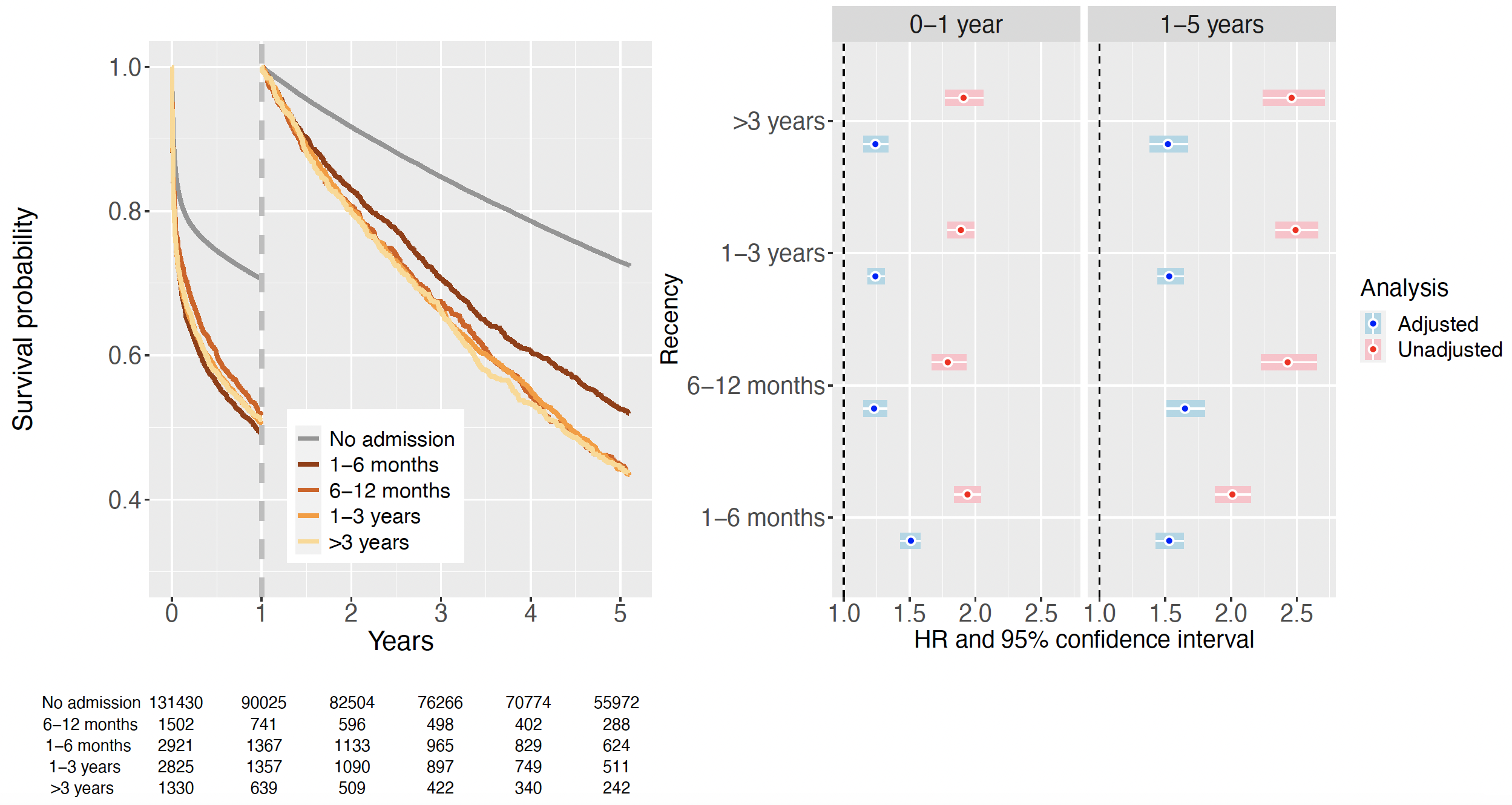


**eFigure S9.** Subgroup analysis restricted to ICU admissions with a SAPS3 reason for admission classified as circulatory (excl. septic shock). Landmark survival analysis described by Kaplan-Meier curves (left panel), stratified for recency of a previous hospital admission with heart failure as the main discharge diagnosis. Hazard ratios (HR) with 95% confidence intervals (CI) (right two panels) have been estimated from Cox proportional hazards models separately for each time period, comparing an unadjusted analysis with an analysis adjusted for sex, age, and other comorbidities.


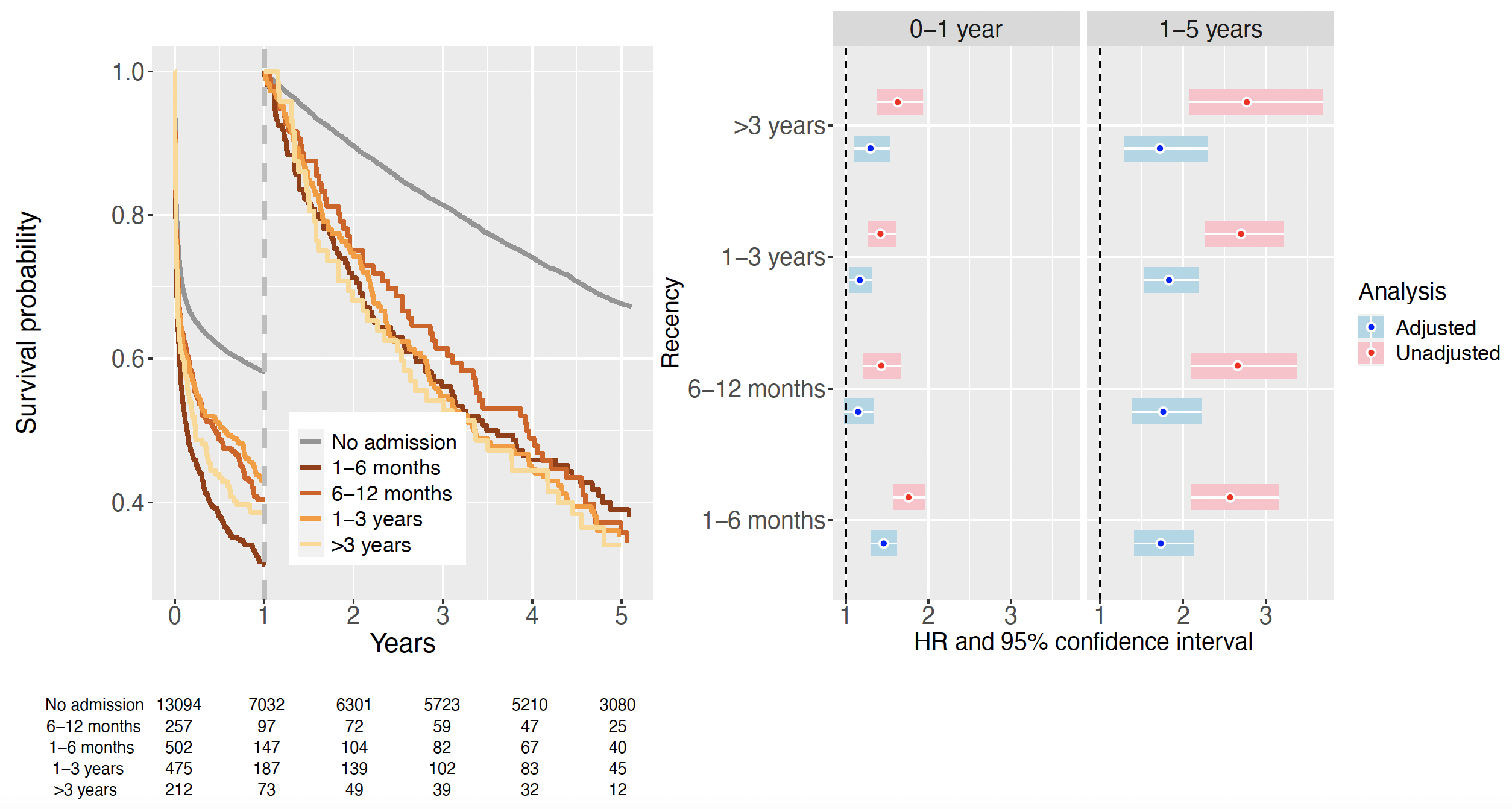


**eFigure S10**. Landmark survival analysis described by Kaplan-Meier curves (left panel), stratified for recency of a previous hospital admission with chronic pulmonary disease as the main discharge diagnosis. Hazard ratios with 95% confidence intervals (right two panels) have been estimated from Cox proportional hazards models separately for each time period, comparing an unadjusted analysis with an analysis adjusted for sex, age, and other comorbidities.


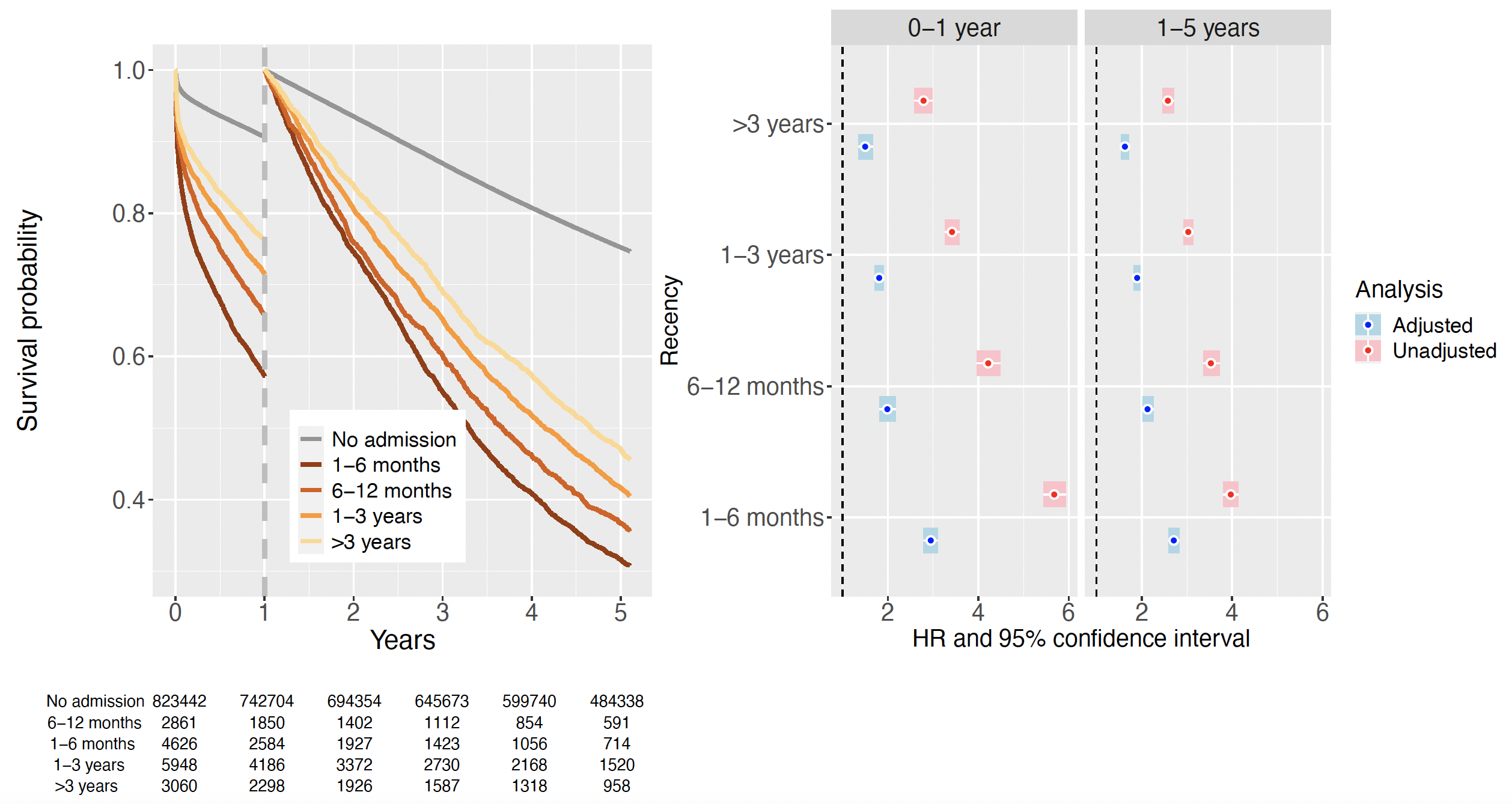


**eFigure S11**. Subgroup analysis restricted to ICU admissions with a SAPS3 reason for admission classified as circulatory (excl. septic shock). Landmark survival analysis described by Kaplan-Meier curves (left panel), stratified for recency of a previous hospital admission with chronic pulmonary disease as the main discharge diagnosis. Hazard ratios (HR) with 95% confidence intervals (CI) (right two panels) have been estimated from Cox proportional hazards models separately for each time period, comparing an unadjusted analysis with an analysis adjusted for sex, age, and other comorbidities.


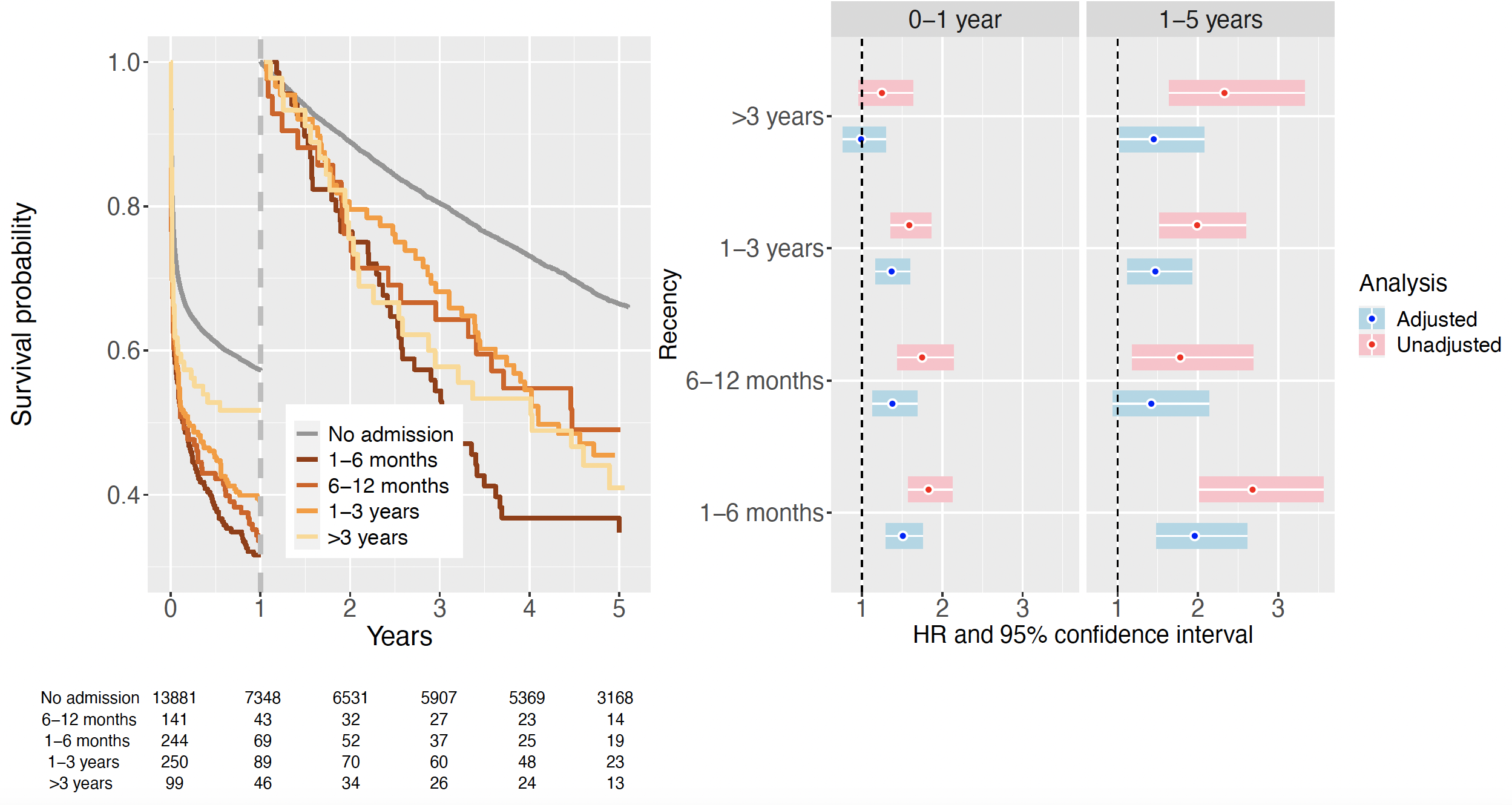


**eFigure S12**. Subgroup analysis restricted to ICU admissions with a SAPS3 reason for admission classified as respiratory, other. Landmark survival analysis described by Kaplan-Meier curves (left panel), stratified for recency of a previous hospital admission with chronic pulmonary disease as the main discharge diagnosis. Hazard ratios (HR) with 95% confidence intervals (CI) (right two panels) have been estimated from Cox proportional hazards models separately for each time period, comparing an unadjusted analysis with an analysis adjusted for sex, age, and other comorbidities.


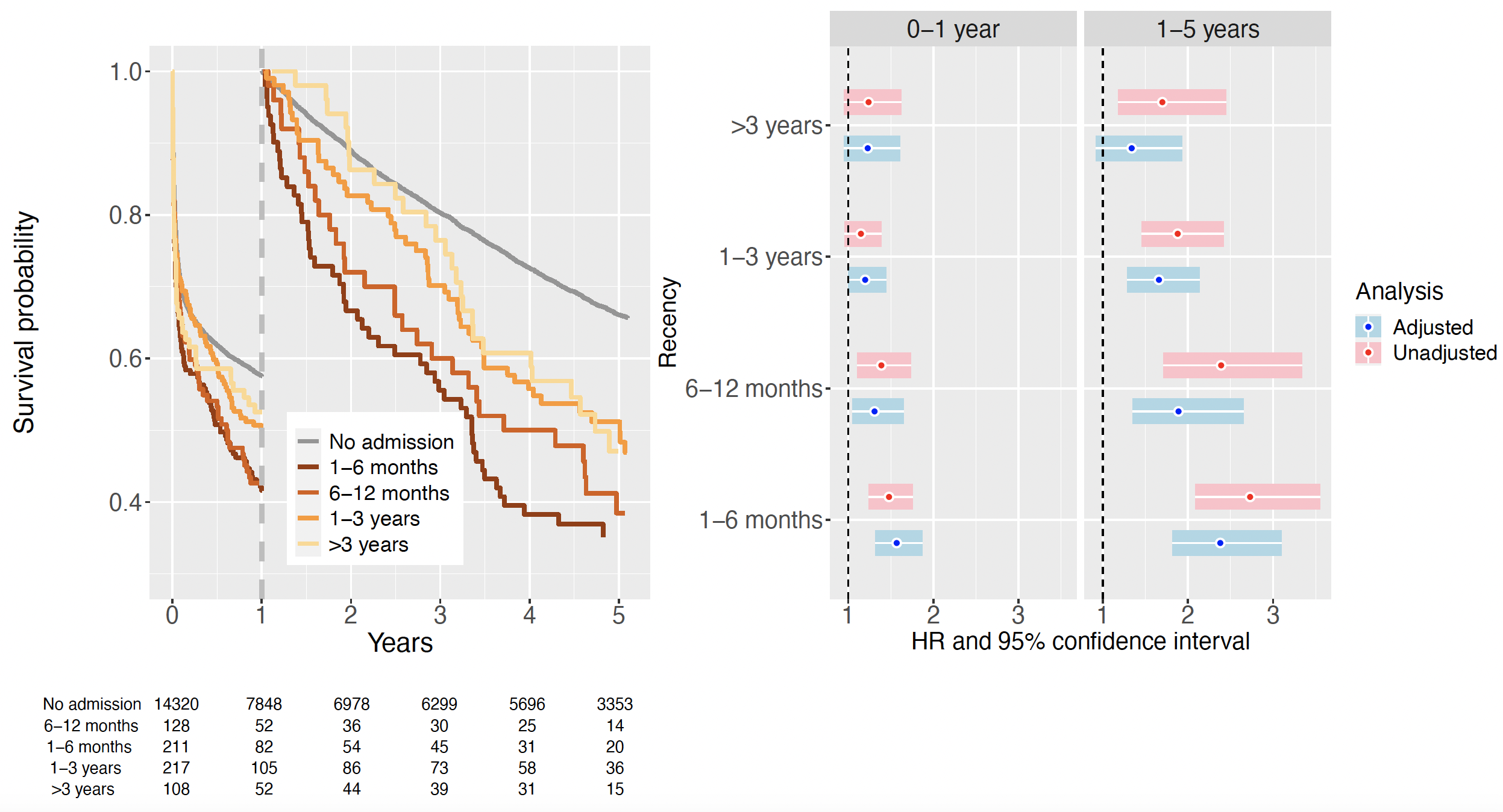


**eTable S6**. Association between recency of single comorbidity, based on hospital admission with a main diagnosis from that category, and survival probability in 140 008 patients ≥55 years old and admitted to an ICU. Hazard ratio (HR) and 95% confidence interval (CI) estimated from Cox proportional hazards regression, unadjusted and adjusted for age, sex, and other comorbidity.

| Main diagnosis category and recency of previous hospital admission used as a measure of comorbidity | Period of follow-up | | | | | | | |
| --- | --- | --- | --- | --- | --- | --- | --- | --- |
|  | 0-1 year | | | | 1-5 years | | | |
|  | Unadjusted | | Adjusted | | Unadjusted | | Adjusted | |
|  | HR | (95% CI) | HR | (95% CI) | HR | (95% CI) | HR | (95% CI) |
| **Previous hospital admission for infectious disease** | | | | | | | | |
| 1-6 months | 1.76 | (1.69 to 1.83) | 1.29 | (1.24 to 1.34) | 2.25 | (2.13 to 2.37) | 1.50 | (1.42 to 1.59) |
| 6-12 months | 1.54 | (1.46 to 1.62) | 1.14 | (1.08 to 1.2) | 2.18 | (2.05 to 2.32) | 1.51 | (1.42 to 1.61) |
| 1-3 years | 1.46 | (1.41 to 1.52) | 1.09 | (1.05 to 1.13) | 2.03 | (1.95 to 2.12) | 1.41 | (1.35 to 1.48) |
| >3 years | 1.33 | (1.26 to 1.39) | 1.06 | (1.01 to 1.11) | 1.78 | (1.69 to 1.89) | 1.36 | (1.28 to 1.43) |
| No previous admission | Reference | | Reference | | Reference | | Reference | |
| **Previous hospital admission for hypertension** | | | | | | | | |
| 1-6 months | 1.19 | (0.97 to 1.45) | 1.05 | (0.86 to 1.28) | 1.49 | (1.19 to 1.88) | 1.18 | (0.94 to 1.49) |
| 6-12 months | 0.95 | (0.75 to 1.2) | 0.87 | (0.69 to 1.1) | 1.36 | (1.08 to 1.73) | 1.10 | (0.87 to 1.39) |
| 1-3 years | 1.11 | (0.98 to 1.26) | 0.93 | (0.82 to 1.05) | 1.48 | (1.29 to 1.69) | 1.16 | (1.01 to 1.33) |
| >3 years | 1.11 | (0.96 to 1.28) | 0.97 | (0.84 to 1.12) | 1.22 | (1.03 to 1.43) | 1.02 | (0.86 to 1.2) |
| No previous admission | Reference | | Reference | | Reference | | Reference | |
| **Previous hospital admission for ischemic heart disease** | | | | | | | | |
| 1-6 months | 1.23 | (1.16 to 1.31) | 1.02 | (0.96 to 1.08) | 1.23 | (1.14 to 1.32) | 0.99 | (0.93 to 1.07) |
| 6-12 months | 1.16 | (1.07 to 1.25) | 0.90 | (0.84 to 0.98) | 1.54 | (1.41 to 1.67) | 1.13 | (1.04 to 1.23) |
| 1-3 years | 1.19 | (1.13 to 1.25) | 0.92 | (0.88 to 0.97) | 1.52 | (1.44 to 1.6) | 1.07 | (1.02 to 1.13) |
| >3 years | 1.15 | (1.09 to 1.21) | 0.92 | (0.87 to 0.97) | 1.41 | (1.32 to 1.5) | 1.04 | (0.98 to 1.1) |
| No previous admission | Reference | | Reference | | Reference | | Reference | |
| **Previous hospital admission for ischemic heart disease. Analysis excluding admissions to thoracic ICUs** | | | | | | | | |
| 1-6 months | 1.23 | (1.16 to 1.31) | 1.02 | (0.96 to 1.08) | 1.23 | (1.14 to 1.32) | 0.99 | (0.93 to 1.07) |
| 6-12 months | 1.16 | (1.07 to 1.25) | 0.9 | (0.84 to 0.98) | 1.54 | (1.41 to 1.67) | 1.13 | (1.04 to 1.23) |
| 1-3 years | 1.19 | (1.13 to 1.25) | 0.92 | (0.88 to 0.97) | 1.52 | (1.44 to 1.6) | 1.07 | (1.02 to 1.13) |
| >3 years | 1.15 | (1.09 to 1.21) | 0.92 | (0.87 to 0.97) | 1.41 | (1.32 to 1.5) | 1.04 | (0.98 to 1.1) |
| No previous admission | Reference | | Reference | | Reference | | Reference | |
| **Previous hospital admission for arrythmia** | | | | | | | | |
| 1-6 months | 1.17 | (1.09 to 1.25) | 1.03 | (0.96 to 1.11) | 1.14 | (1.06 to 1.24) | 0.99 | (0.92 to 1.08) |
| 6-12 months | 1.16 | (1.07 to 1.26) | 0.96 | (0.89 to 1.04) | 1.26 | (1.15 to 1.38) | 0.99 | (0.91 to 1.08) |
| 1-3 years | 1.30 | (1.23 to 1.37) | 0.95 | (0.91 to 1) | 1.53 | (1.45 to 1.62) | 1.07 | (1.01 to 1.13) |
| >3 years | 1.43 | (1.35 to 1.51) | 1.04 | (0.98 to 1.11) | 1.56 | (1.46 to 1.68) | 1.04 | (0.97 to 1.12) |
| No previous admission | Reference | | Reference | | Reference | | Reference | |
| **Previous hospital admission for heart failure** | | | | | | | | |
| 1-6 months | 2.08 | (1.98 to 2.19) | 1.51 | (1.43 to 1.59) | 2.57 | (2.38 to 2.78) | 1.61 | (1.49 to 1.73) |
| 6-12 months | 1.78 | (1.66 to 1.92) | 1.23 | (1.14 to 1.32) | 2.66 | (2.41 to 2.92) | 1.60 | (1.46 to 1.76) |
| 1-3 years | 1.74 | (1.65 to 1.84) | 1.22 | (1.15 to 1.28) | 2.44 | (2.28 to 2.61) | 1.49 | (1.4 to 1.6) |
| >3 years | 1.76 | (1.63 to 1.9) | 1.22 | (1.13 to 1.32) | 2.45 | (2.22 to 2.7) | 1.51 | (1.37 to 1.66) |
| No previous admission | Reference | | Reference | | Reference | | Reference | |
| **Previous hospital admission for heart failure. Analysis restricted to subgroup with cardiovascular reason for ICU admission** | | | | | | | | |
| 1-6 months | 1.76 | (1.58 to 1.96) | 1.46 | (1.31 to 1.62) | 2.57 | (2.1 to 3.15) | 1.73 | (1.41 to 2.13) |
| 6-12 months | 1.43 | (1.22 to 1.67) | 1.15 | (0.98 to 1.34) | 2.66 | (2.1 to 3.38) | 1.76 | (1.38 to 2.23) |
| 1-3 years | 1.42 | (1.27 to 1.6) | 1.17 | (1.04 to 1.32) | 2.70 | (2.26 to 3.22) | 1.83 | (1.53 to 2.19) |
| >3 years | 1.63 | (1.38 to 1.93) | 1.30 | (1.1 to 1.54) | 2.77 | (2.08 to 3.69) | 1.72 | (1.29 to 2.3) |
| No previous admission | Reference | | Reference | | Reference | | Reference | |
| **Previous hospital admission for heart failure. Analysis restricted to subgroup with respiratory (other than acute-on-chronic) reason for ICU admission** | | | | | | | | |
| 1-6 months | 1.97 | (1.72 to 2.25) | 1.54 | (1.35 to 1.77) | 2.35 | (1.82 to 3.05) | 1.53 | (1.18 to 1.98) |
| 6-12 months | 1.58 | (1.29 to 1.93) | 1.19 | (0.97 to 1.45) | 2.76 | (2.05 to 3.74) | 1.72 | (1.27 to 2.33) |
| 1-3 years | 1.44 | (1.25 to 1.66) | 1.08 | (0.93 to 1.24) | 2.46 | (2.00 to 3.01) | 1.44 | (1.17 to 1.77) |
| >3 years | 1.67 | (1.37 to 2.04) | 1.27 | (1.04 to 1.55) | 2.99 | (2.20 to 4.05) | 1.95 | (1.44 to 2.65) |
| No previous admission | Reference | | Reference | | Reference | | Reference | |
| **Previous hospital admission for chronic pulmonary disease** | | | | | | | | |
| 1-6 months | 1.84 | (1.74 to 1.93) | 1.59 | (1.51 to 1.68) | 3.11 | (2.90 to 3.34) | 2.64 | (2.46 to 2.83) |
| 6-12 months | 1.68 | (1.55 to 1.81) | 1.39 | (1.28 to 1.5) | 2.71 | (2.45 to 2.98) | 2.12 | (1.92 to 2.34) |
| 1-3 years | 1.54 | (1.45 to 1.63) | 1.33 | (1.25 to 1.41) | 2.27 | (2.11 to 2.43) | 1.82 | (1.69 to 1.95) |
| >3 years | 1.42 | (1.3 to 1.56) | 1.22 | (1.12 to 1.34) | 2.03 | (1.83 to 2.26) | 1.58 | (1.42 to 1.76) |
| No previous admission | Reference | | Reference | | Reference | | Reference | |
| **Previous hospital admission for chronic pulmonary disease. Analysis restricted to subgroup with cardiovascular reason for ICU admission** | | | | | | | | |
| 1-6 months | 1.82 | (1.57 to 2.12) | 1.61 | (1.39 to 1.88) | 2.67 | (2.01 to 3.54) | 1.91 | (1.43 to 2.54) |
| 6-12 months | 1.75 | (1.43 to 2.13) | 1.62 | (1.32 to 1.98) | 1.78 | (1.18 to 2.68) | 1.41 | (0.93 to 2.13) |
| 1-3 years | 1.58 | (1.35 to 1.85) | 1.36 | (1.16 to 1.59) | 1.98 | (1.51 to 2.59) | 1.43 | (1.09 to 1.88) |
| >3 years | 1.25 | (0.95 to 1.63) | 1.06 | (0.81 to 1.39) | 2.32 | (1.63 to 3.31) | 1.41 | (0.99 to 2.01) |
| No previous admission | Reference | | Reference | | Reference | | Reference | |
| **Previous hospital admission for chronic pulmonary disease. Analysis restricted to subgroup with respiratory (other than acute-on-chronic) reason for ICU admission** | | | | | | | | |
| 1-6 months | 1.48 | (1.24 to 1.76) | 1.22 | (1.03 to 1.46) | 2.72 | (2.09 to 3.55) | 2.21 | (1.69 to 2.88) |
| 6-12 months | 1.39 | (1.11 to 1.74) | 1.17 | (0.93 to 1.47) | 2.39 | (1.71 to 3.34) | 1.86 | (1.33 to 2.6) |
| 1-3 years | 1.15 | (0.96 to 1.39) | 1.03 | (0.85 to 1.24) | 1.88 | (1.46 to 2.42) | 1.56 | (1.21 to 2.01) |
| >3 years | 1.24 | (0.95 to 1.62) | 1.07 | (0.82 to 1.39) | 1.70 | (1.18 to 2.45) | 1.33 | (0.92 to 1.92) |
| No previous admission | Reference | | Reference | | Reference | | Reference | |

**eTable S7.** Comparison of characteristics between ICU patients with and without SAPS3 score registered. SAPS3 score only available from the year 2009 in the Swedish Intensive Care register.

|  | N | SAPS3 available  (N=43 685) | SAPS3 missing  (N=34 368) |
| --- | --- | --- | --- |
| **Age** (years), median (interquartile range) | 78 053 | 72 (65 - 80) | 70 (64 - 78) |
| **Age** (years), % (n) | 78 053 |  |  |
| <60 |  | 10% (4366) | 11% (3949) |
| 60-69 |  | 32% (13 856) | 35% (12 045) |
| 70-79 |  | 33% (14 405) | 34% (11 616) |
| 80-89 |  | 23% (9921) | 18% (6155) |
| ≥90 |  | 3% (1137) | 2% (603) |
| **Sex** | 78 053 |  |  |
| Female |  | 43% (18 699) | 38% (13 158) |
| **Calendar year** | 78 053 |  |  |
| 2009 |  | 13% (5628) | 36% (12 321) |
| 2010 |  | 24% (10 568) | 26% (9013) |
| 2011 |  | 28% (12 282) | 23% (7872) |
| 2012 |  | 35% (15 207) | 15% (5162) |
| **ICU type** | 78 053 |  |  |
| Thoracic |  | 0% (81) | 34% (11 789) |
| General |  | 100% (43 604) | 66% (22 579) |
| **ICU length of stay** | 78 053 |  |  |
| <1 day |  | 20% (8754) | 18% (6236) |
| 1-2 days |  | 51% (22 469) | 61% (21 121) |
| 3-6 days |  | 18% (7764) | 14% (4699) |
| 7-13 days |  | 7% (2843) | 4% (1458) |
| ≥14 days |  | 4% (1855) | 2% (854) |
| **Type of comorbidity** |  |  |  |
| Hypertension | 78 053 | 31% (13 572) | 29% (10 075) |
| Ischemic heart disease | 78 053 | 16% (6994) | 20% (6805) |
| Infectious disease | 78 053 | 24% (10 499) | 18% (6124) |
| Cardiac arrhythmias | 78 053 | 17% (7399) | 15% (5287) |
| Diabetes | 78 053 | 15% (6607) | 13% (4513) |
| Bone/muscle disease | 78 053 | 15% (6343) | 12% (4254) |
| Congestive heart failure | 78 053 | 13% (5749) | 11% (3901) |
| Injury | 78 053 | 13% (5861) | 11% (3707) |
| Neurological disease | 78 053 | 14% (6006) | 11% (3686) |
| Valvular disease | 78 053 | 3% (1360) | 11% (3663) |
| Tumour non-metastatic | 78 053 | 11% (4592) | 9% (3252) |
| Chronic pulmonary disease | 78 053 | 13% (5605) | 9% (3086) |
| Cerebrovascular disease | 78 053 | 9% (4103) | 8% (2621) |
| Peripheral vascular disease | 78 053 | 7% (3271) | 7% (2415) |
| Other anemias | 78 053 | 8% (3620) | 6% (2073) |
| Renal disease | 78 053 | 7% (3010) | 5% (1742) |
| Other endocrine disease | 78 053 | 5% (2360) | 4% (1520) |
| Alcohol abuse | 78 053 | 5% (2091) | 4% (1267) |
| Rheumatic/autoimmune disease | 78 053 | 4% (1853) | 3% (1170) |
| Depression | 78 053 | 4% (1622) | 3% (1054) |
| Fluid balance disorder | 78 053 | 5% (2042) | 3% (1031) |
| Drug abuse | 78 053 | 2% (825) | 2% (742) |
| Hepatic disease | 78 053 | 3% (1129) | 2% (694) |
| Tumour metastatic | 78 053 | 2% (954) | 2% (637) |
| Pulmonary circulation disorders | 78 053 | 2% (1004) | 2% (598) |
| Hematological disease | 78 053 | 2% (850) | 1% (474) |
| Hematological malignancy | 78 053 | 2% (909) | 1% (462) |
| Obesity | 78 053 | 2% (771) | 1% (462) |
| Poisoning | 78 053 | 1% (607) | 1% (435) |
| Blood loss anemia | 78 053 | 1% (530) | 1% (320) |
| Psychoses | 78 053 | 1% (545) | 1% (288) |
| Malnutrition | 78 053 | 1% (283) | 1% (182) |
| Coagulopathy | 78 053 | 1% (226) | 1% (176) |
| Immunodeficiency | 78 053 | 0% (74) | 0% (44) |
| Transplantation-related disorder | 78 053 | 1% (228) | 0% (166) |

**eFigure S13**. Survival probability of ICU patients with and without SAPS3 score registered. SAPS3 score is only available from the year 2009 in the Swedish Intensive Care register. The analysis is therefore restricted to ICU admissions during the years 2009-2013.


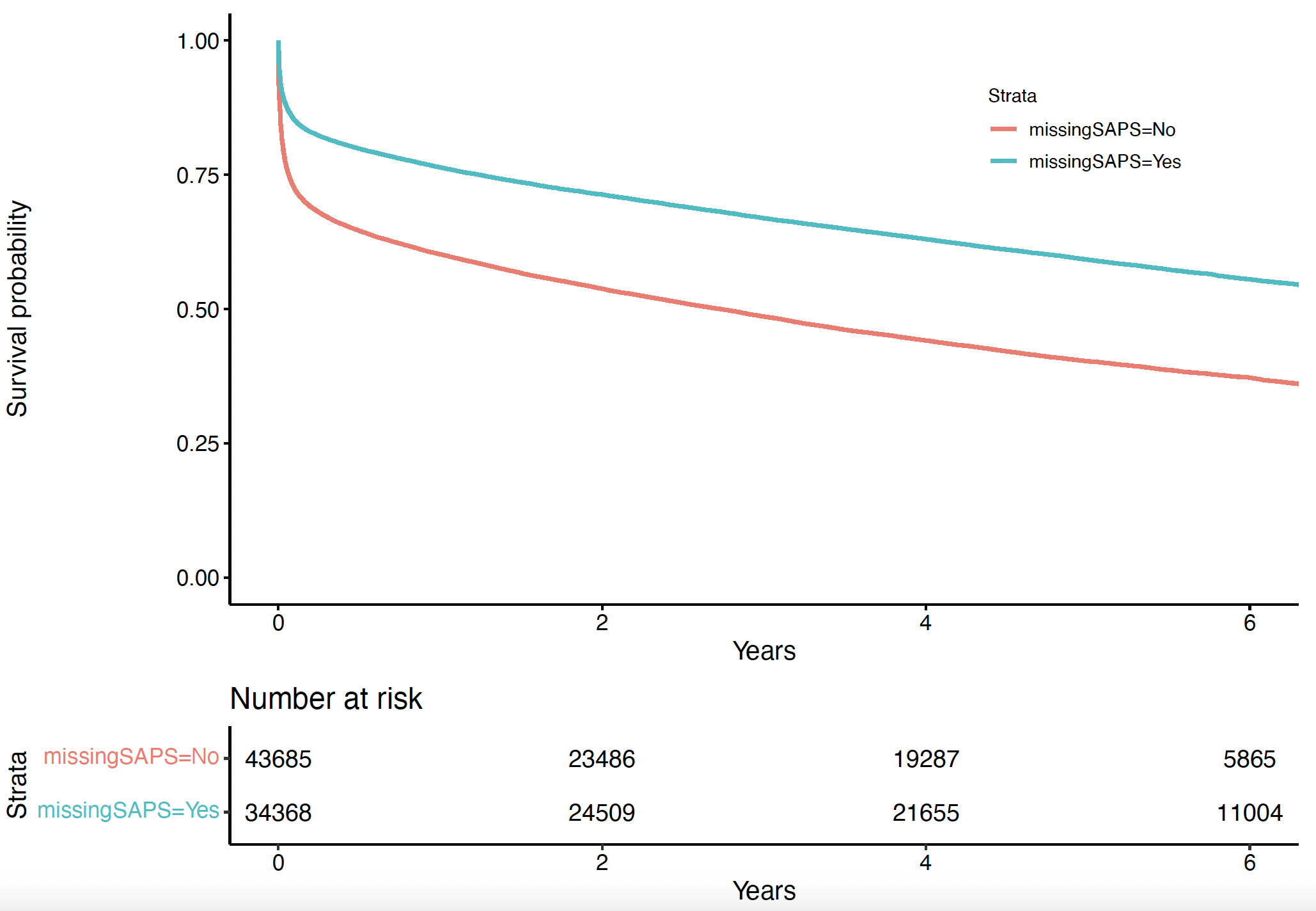


**eFigure S14**. Diagnostics for the overlap of the propensity score distributions in the main analysis Cox regression for the entire study population.

**
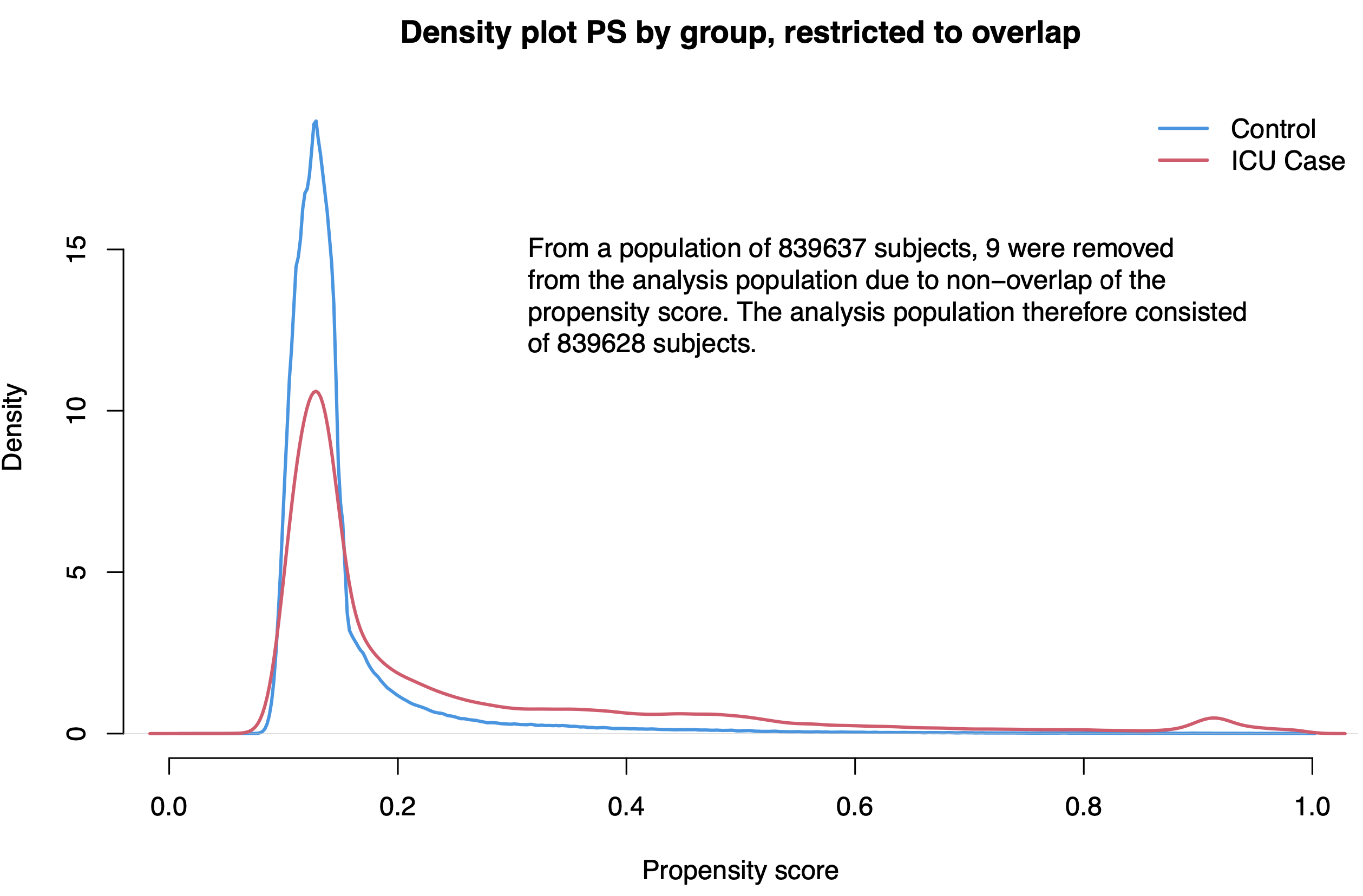
**

**eFigure S15**. Diagnostics for the proportional hazards assumption for the Cox regression analysis of the main comparison between ICU patients and matched comparator subjects from the general population. The analysis is for the period after the 1-year landmark and adjusted for covariates as indicated in the main results. The *upper panel* presents an estimate of the time-dependent coefficient β(t) for the Schoenfeld residuals over time, which is expected to be a horizontal line if the proportional hazards assumption holds true. The p-values are from the test for slope = 0 (for the group variable and a global test from R function cox.zph). The *lower panel* provides a -log-log plot.


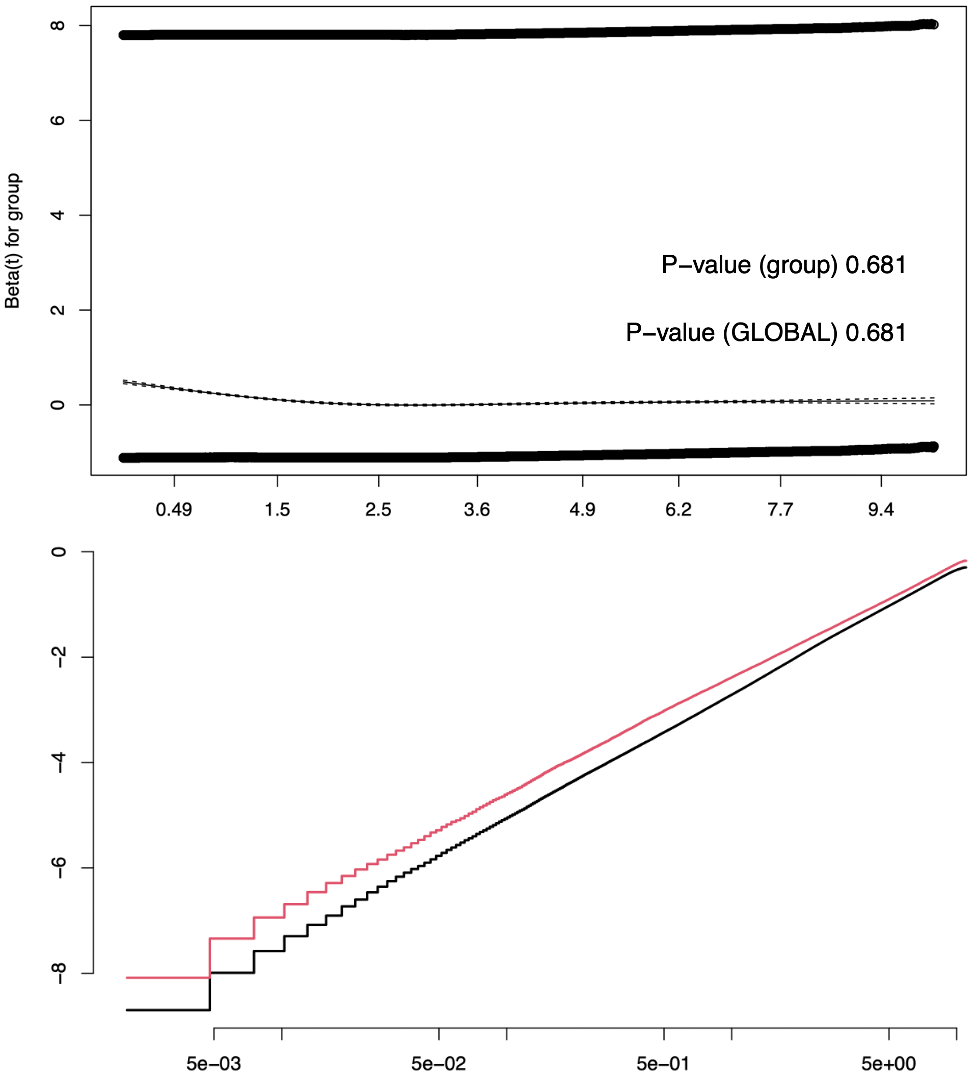

Supplement: Supplementary file 1 — Supplementary Material 1. [file 13054_2024_5147_MOESM1_ESM.docx]
